# HIF-2α in Lung Biology and Disease: From Adaptation and Repair to Therapeutic Modulation

**DOI:** 10.3390/biology15211907

**Published:** 2026-10-22

**Authors:** Hon-Sum Jeffrey Man, Aninda Dibya Saha, Licun Wu, Marc de Perrot

**Affiliations:** 1 Departments of Respirology and Critical Care Medicine, University Health Network and Sinai Health System; Division of Respirology and Interdepartmental Division of Critical Care Medicine, University of Toronto, Toronto, ON M5G 2C4, Canada; aninda.saha@alumni.utoronto.ca; 2 Latner Thoracic Research Laboratories, Toronto General Hospital Research Institute, University of Toronto, Toronto, ON M5G 1L7, Canada; licun.wu1@uhn.ca; 3 Department of Immunology, University of Toronto, Toronto, ON M5S 1A8, Canada; 4 Division of Thoracic Surgery, University Health Network, University of Toronto, Toronto, ON M5G 2C4, Canada

**Keywords:** HIF-2α, *EPAS1*, hypoxia-inducible factor, oxygen sensing, lung disease, pulmonary hypertension, pulmonary fibrosis, COPD, acute respiratory distress syndrome, long noncoding RNA

## Abstract

Hypoxia-inducible factor 2α (HIF-2α), encoded by *EPAS1*, has long been studied in erythropoiesis, high-altitude adaptation, and cancer. Recent studies also implicate HIF-2α within non-malignant lung development and disease. This review examines HIF-2α as a dosage-sensitive, context-dependent regulator of lung cell state rather than a uniformly protective or pathogenic transcription factor. HIF-2α inhibitors have been approved in oncology, while pulmonary studies demonstrate roles for HIF-2α in fetal lung maturation, endothelial barrier integrity, alveolar–capillary maintenance, pulmonary vascular remodeling, aberrant epithelial repair, fibroblast persistence, airway differentiation, and alveolar macrophage metabolic and surfactant homeostasis. We summarize the canonical *EGLN*/PHD–VHL–HIF pathway, the HIF-1α/HIF-2α isoform bias, human genetic evidence from chronic hypoxia adaptation, and emerging disease-specific data in pulmonary arterial hypertension, pulmonary fibrosis, COPD/emphysema, acute lung injury/ARDS, and muco-obstructive disease. We also highlight chromatin, long noncoding RNA, and post-transcriptional mechanisms that tune HIF-2α activity. Together, these findings support the need to precisely tailor HIF-2α modulation in lung disease, guided by cell type, disease phase, spatial context, and biological objective.

## Simple Summary

The lungs are continuously exposed to changing oxygen levels, and their cells rely on oxygen-sensing pathways to maintain normal function and respond to injury. Hypoxia-inducible factor 2 alpha is a key component of this system. It is already a proven drug target in cancer. Recent studies suggest that it also has important and sometimes opposing roles in lung health and disease. In some settings, hypoxia-inducible factor 2 alpha supports lung development, protects blood vessel barriers, and helps maintain the delicate interface where oxygen passes from the lungs into the blood. In others, persistent or misplaced activity may contribute to pulmonary hypertension, lung scarring, abnormal repair, and changes in immune-cell function. Human studies of adaptation to life at high altitude also show that this pathway can be finely adjusted rather than simply turned on or off. This review brings these findings together and considers how they could guide future treatments. The evidence suggests that successful therapies may need to account for which lung cells are involved and whether the goal is to reduce harmful activity or preserve beneficial functions.

## 1. Introduction: Why HIF-2α Matters Beyond Oncology

The rise of atmospheric oxygen approximately 2.4 billion years ago during the Great Oxidation Event provided the energetic foundation for complex multicellular organisms while creating the challenge of maintaining oxygen homeostasis across diverse tissues [1,2]. The evolution of sophisticated oxygen-sensing mechanisms therefore became a prerequisite for multicellular life, allowing organisms to couple oxygen availability to metabolism, growth, vascular development, and tissue repair. Central to this adaptation is the hypoxia-inducible factor (HIF) pathway, an evolutionarily conserved transcriptional system that enables cells to continuously monitor oxygen availability and coordinate appropriate genetic responses [3,4].

Importantly, oxygen sensing is not simply a response to extreme hypoxia. Although ambient inspired air contains approximately 21% O_2_, physiological tissue oxygen tensions are substantially lower and vary widely across organs and microenvironments, with some physiological niches approaching 1–3% or less, including regions of the bone marrow, kidney, and stem cell microenvironments [5]. Even within the lung—the organ responsible for oxygen exchange—steep oxygen gradients exist between the airspaces, conducting airways, pulmonary vasculature, and injured or remodeling tissues. Consequently, HIF signaling functions not merely as an emergency response to oxygen deprivation, but as a dynamic regulator that continuously integrates oxygen availability with developmental, inflammatory, metabolic, and mechanical signals to determine cell behavior in both health and disease [3,6].

Since the discovery of HIF-1α in the mid-1990s [7,8], our understanding of oxygen sensing has evolved from a relatively simple pathway controlling erythropoietin production into one of the central regulatory systems governing mammalian physiology. HIF signaling now encompasses developmental patterning, angiogenesis, vascular homeostasis, immune regulation, metabolism, stem cell biology, tissue regeneration, and adaptation to environmental stress. Unsurprisingly, dysregulated HIF activity has been implicated across a diverse spectrum of human diseases, including cancer, cardiovascular disease, pulmonary hypertension, chronic lung disease, fibrosis, inflammatory disorders, and anemia [3,4].

**Figure 1:**
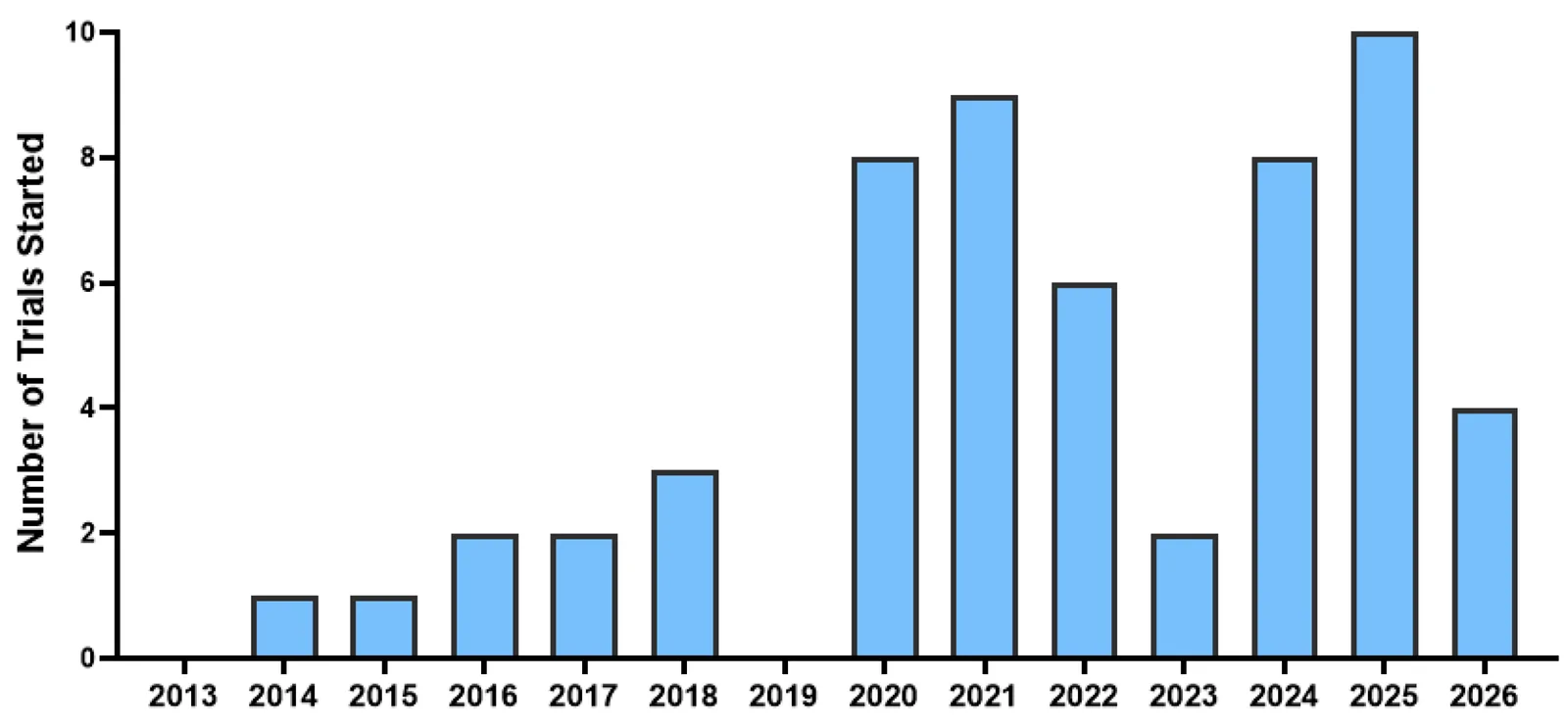
Frequency histogram of clinical trials of HIF-2α inhibitors by year.

Among the oxygen-regulated HIF-α isoforms, HIF-2α, encoded by *EPAS1*, has emerged as a particularly compelling therapeutic target. Initially recognized for its roles in vascular biology, erythropoiesis, and chronic adaptation to hypoxia [9,10], HIF-2α is now understood to regulate endothelial homeostasis, progenitor cell maintenance, immune function, tissue remodeling, and cell-state transitions across multiple organs. Once considered “undruggable,” HIF-2α became one of the first transcription factors to be successfully targeted through structure-guided drug design [11] following the discovery of a ligand-binding cavity within its PAS-B domain [12,13,14]. The subsequent clinical development of belzutifan has established proof-of-principle that selective pharmacological modulation of HIF-2α is both feasible and therapeutically effective in patients with von Hippel–Lindau disease and advanced clear cell renal cell carcinoma (Figure 1) (Tables S1 and S2) [15,16,17]. More recently, HIF-2α inhibition has also been shown to rapidly reverse cancer-associated cachexia and metabolic dysfunction, illustrating that modulation of this pathway can reshape systemic physiology beyond direct anti-tumor effects [18].

These advances raise an exciting question of where HIF-2α-directed therapies should be applied next. The lung is a promising and biologically complex target for therapeutic manipulation of oxygen sensing. Experimental and human studies now implicate HIF-2α in pulmonary vascular remodeling [19,20,21], emphysema [22], fibrosis and epithelial regeneration [23], acute lung injury [24], and immune cell differentiation [25]. Yet these studies also reveal an important distinction from oncology. Whereas HIF-2α frequently acts as a pathogenic driver in renal cell carcinoma, its functions within the lung are profoundly context-dependent. Depending on the cell type, developmental stage, and disease state, HIF-2α may preserve endothelial integrity, support epithelial repair, regulate immune cell maturation, or alternatively promote pathological remodeling. Thus, both excessive and insufficient HIF-2α activity may contribute to pulmonary disease.

This emerging complexity suggests that the future of HIF-2α therapeutics in pulmonary medicine lies in precision modulation: tailoring the timing, magnitude, and cellular specificity of intervention to the underlying biology. Human genetic studies further reinforce this concept, demonstrating that relatively modest alterations in HIF pathway activity can produce profound physiological adaptations to hypoxia without overt pathology, highlighting the pathway’s exquisite dosage sensitivity [26,27,28].

In this Review, we first provide a concise overview of canonical oxygen sensing, HIF biology, and insights from human hypoxia genetics. We then synthesize recent advances defining the diverse and cell-specific functions of HIF-2α across the pulmonary vasculature, epithelium, immune system, and stromal compartments in health and disease. Finally, we discuss how these biological principles should inform the next generation of HIF-directed therapeutics, arguing that successful translation to pulmonary disease will likely require moving beyond uniform pathway inhibition toward increasingly precise modulation of oxygen-sensing networks according to cellular context, disease stage, and therapeutic objective.

## 2. Overview of the EGLN/PHD–VHL–HIF Pathway and the HIF-1α/HIF-2α Switch

**Figure 2:**
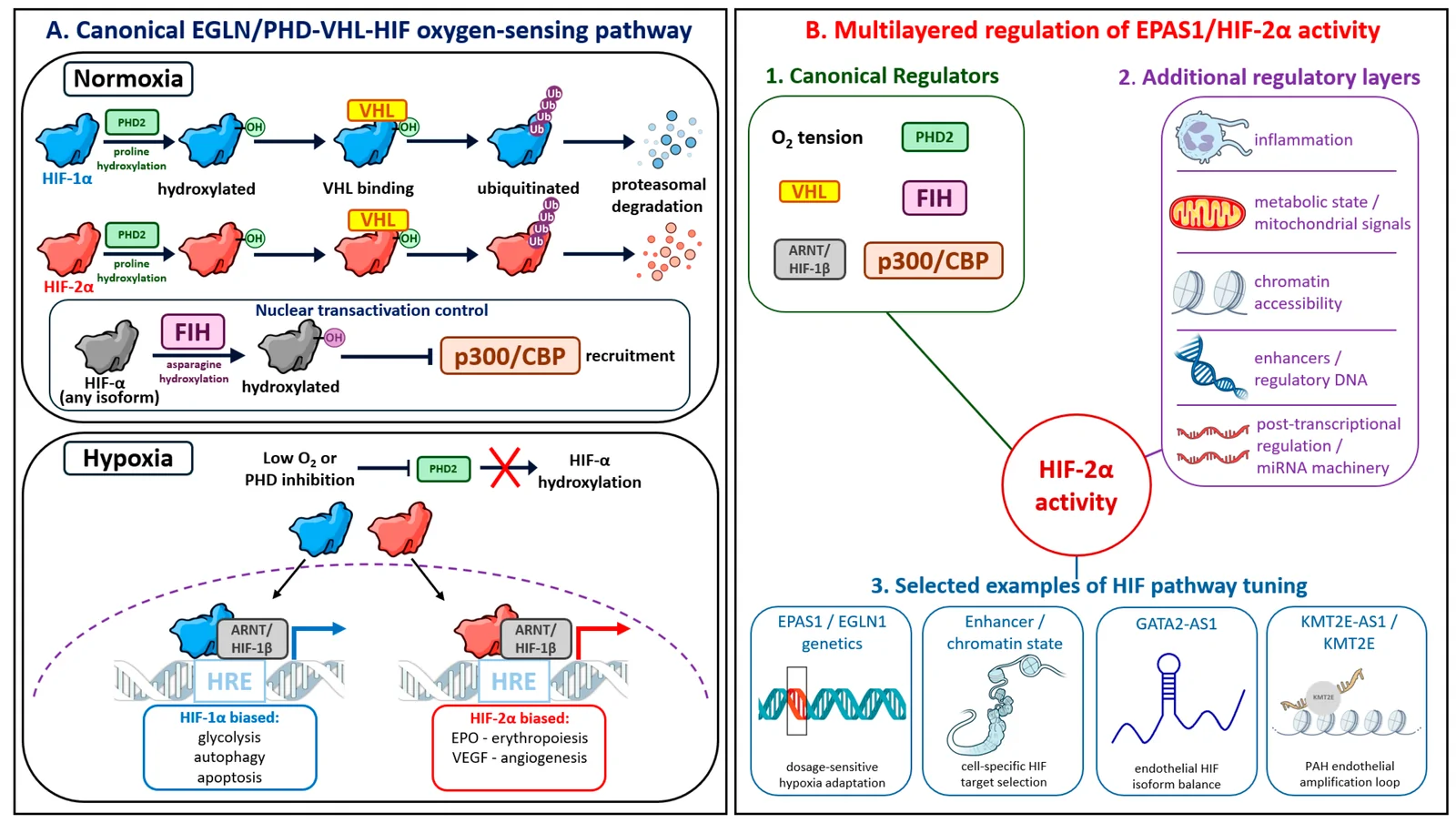
Canonical oxygen sensing, HIF-α isoform bias, and multilayered regulation of HIF-2α activity. (**A**) The canonical PHD–VHL–HIF pathway regulates HIF-α protein stability and transcriptional activity according to oxygen availability. Under normoxia, oxygen-dependent prolyl hydroxylase domain enzymes, particularly PHD2, hydroxylate HIF-α proteins on conserved proline residues. Hydroxylated HIF-1α and HIF-2α are recognized by the hydroxyproline-binding pocket of the von Hippel–Lindau (VHL) E3 ubiquitin ligase complex, ubiquitinated, and targeted for proteasomal degradation. A second oxygen-dependent hydroxylase, factor inhibiting HIF-1 (FIH/HIF1AN), hydroxylates the HIF-α C-terminal transactivation domain and limits p300/CBP coactivator recruitment, thereby restraining HIF transcriptional activity even when HIF-α protein is present. Under hypoxia, PHD2 activity decreases, HIF-α hydroxylation is reduced, and HIF-1α and HIF-2α escape efficient VHL recognition. Stabilized HIF-1α and HIF-2α translocate to the nucleus, where each dimerizes with HIF-1β at hypoxia-response elements (HREs) and activates partially overlapping but isoform-biased transcriptional programs. HIF-1α-biased outputs include glycolysis and acute cellular survival, whereas HIF-2α-biased outputs include EPO, VEGF, and vascular/endothelial programs. (**B**) HIF-2α activity is regulated by canonical oxygen-sensing components and by additional cell-state-specific regulatory layers. Canonical regulators include oxygen tension, PHD activity, VHL, FIH/HIF1AN, HIF-1β, and p300/CBP. Additional regulatory inputs include inflammatory signaling, metabolic and mitochondrial state, chromatin accessibility, enhancers and regulatory DNA, post-transcriptional control, long noncoding RNAs, and miRNA-processing machinery. Selected examples of HIF-pathway tuning include *EPAS1*/*EGLN1* genetic variation in chronic hypoxia adaptation, enhancer and chromatin state as determinants of cell-specific HIF target selection, *GATA2-AS1* as an endothelial regulator of HIF isoform balance, and the *KMT2E-AS1*/KMT2E axis as a HIF-2α-associated endothelial amplification loop in pulmonary arterial hypertension. Together, these regulatory layers suggest that therapeutic modulation of HIF-2α in lung disease may require indirect and cell-specific strategies, including regulatory RNA targeting, downstream circuit modulation, or targeted delivery, rather than global activation or inhibition of the pathway.

The canonical oxygen-sensing pathway is summarized in Figure 2. HIF transcription factors are heterodimers composed of an oxygen-regulated α-subunit (HIF-α) and a constitutively expressed β-subunit, aryl hydrocarbon receptor nuclear translocator/HIF-1β (ARNT/HIF-1β). Together, these proteins bind hypoxia-response elements (HREs) and regulate genes involved in metabolism, angiogenesis, erythropoiesis, cell survival, proliferation, and tissue adaptation [3,4,7]. Under oxygen-replete conditions, HIF-α subunits are hydroxylated on conserved proline residues by *EGLN*/PHD enzymes, particularly *EGLN1*/PHD2. Hydroxylated HIF-α is recognized by the VHL E3 ubiquitin ligase complex and targeted for proteasomal degradation. When oxygen availability falls, *EGLN*/PHD activity decreases, HIF-α proteins escape efficient VHL-mediated degradation, accumulate, dimerize with ARNT/HIF-1β, and activate hypoxia-responsive transcriptional programs [4,29,30].

Protein stabilization, however, is only one determinant of HIF activity. Transcriptional output is further shaped by oxygen-dependent regulators such as factor inhibiting HIF-1 (FIH/HIF1AN), which limits co-activator recruitment, together with oxygen-independent influences including inflammatory and growth-factor signaling, metabolic and redox state, chromatin accessibility, and feedback within the PHD–VHL axis [31,32,33]. Consequently, apparent HIF activation in lung disease may reflect true hypoxia, pseudohypoxic signaling, or altered pathway regulation rather than oxygen deprivation alone [34].

**Figure 3:**
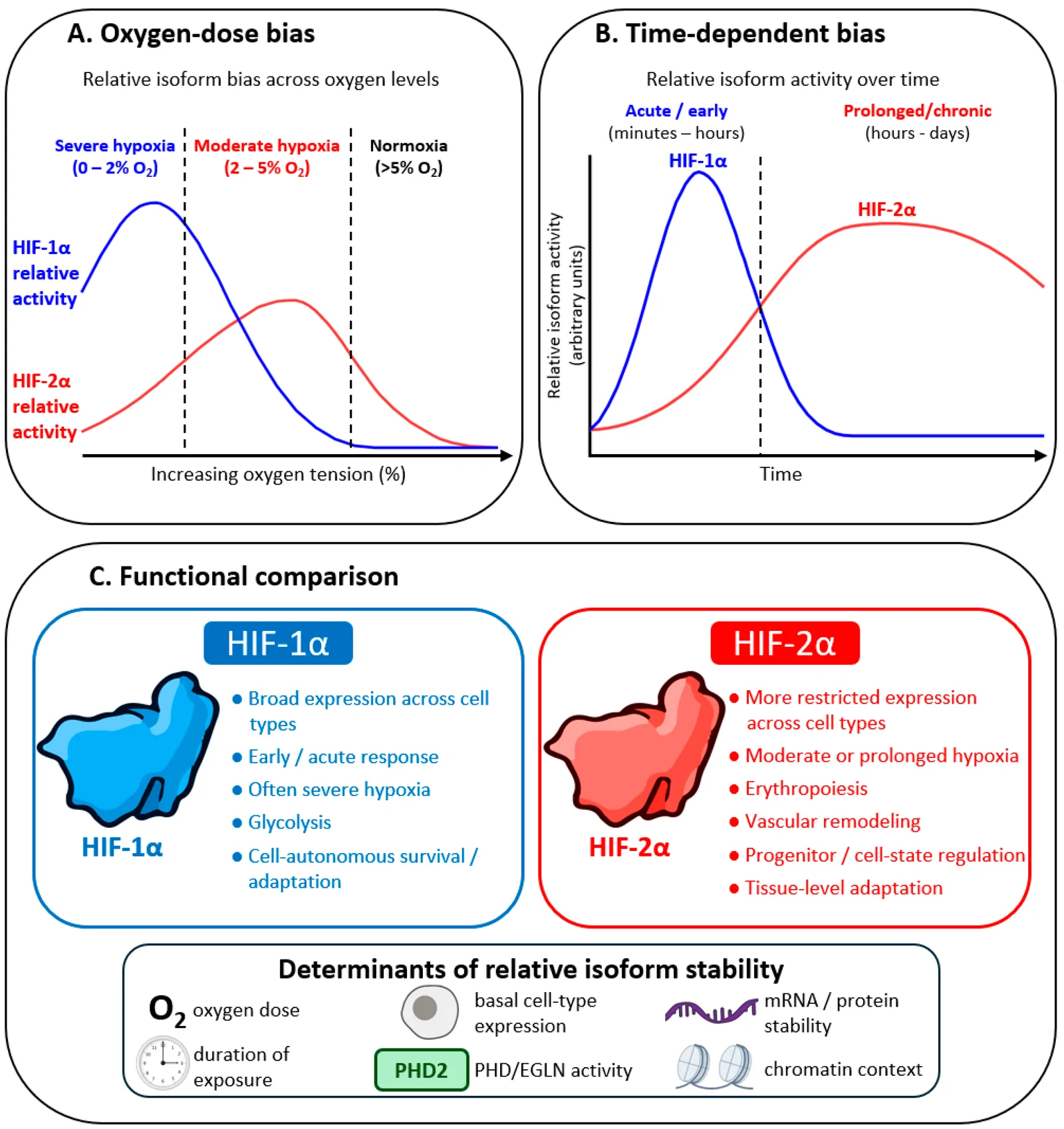
HIF-1α versus HIF-2α: oxygen-dose and time-dependent bias in hypoxia-inducible factor pathway output. Schematic summary of the general differences between hypoxia-inducible factor 1α (HIF-1α) and hypoxia-inducible factor 2α (HIF-2α). (**A**) HIF-1α is typically favored under severe hypoxia, whereas HIF-2α is more often associated with moderate hypoxia. (**B**) HIF-1α commonly rises early during acute hypoxic exposure and then declines, while HIF-2α is often more sustained during prolonged or chronic hypoxia. (**C**) HIF-1α is broadly expressed across cell types and is associated with glycolysis and acute cell-autonomous adaptation. HIF-2α has more restricted expression across cell types and is linked to endothelial biology, erythropoiesis, vascular remodeling, progenitor or cell-state regulation, and tissue-level adaptation. Relative isoform activity is shaped by oxygen dose, duration of exposure, basal cell-type expression, Egl-9 family hypoxia-inducible factor/prolyl hydroxylase domain enzyme (*EGLN*/PHD) activity, messenger ribonucleic acid (mRNA) and protein stability, and chromatin context. These patterns should be interpreted as conceptual biases rather than absolute rules. Adapted from [10,35,36,37,38,39,40,41].

HIF-1α and HIF-2α share this core regulatory architecture, but they are not interchangeable (Figure 3). HIF-1α is broadly expressed and is often the dominant mediator of acute cellular adaptation to severe hypoxia, including glycolytic reprogramming and short-term survival responses. HIF-2α, encoded by *EPAS1*, has a more restricted distribution and is enriched in selected cell populations, including endothelial cells, hepatocytes, interstitial cells, and macrophage-lineage populations. It has prominent roles in erythropoiesis, endothelial biology, vascular remodeling, and chronic hypoxic adaptation [10,35,36]. A useful way to think about the two isoforms is that HIF-1α primarily supports acute, cell-autonomous adaptation, whereas HIF-2α more often coordinates sustained tissue-level responses involving vascular, erythropoietic, epithelial, immune, and stromal programs.

The frequently cited “HIF switch” between HIF-1α and HIF-2α is best viewed as a shift in pathway bias rather than a simple handoff between redundant isoforms (Figure 3). The relative contribution of each isoform depends on oxygen concentration, duration of hypoxic exposure, intrinsic cell type, and the surrounding regulatory environment. In many experimental systems, HIF-1α accumulates rapidly during acute hypoxia before declining with prolonged exposure, whereas HIF-2α is often stabilized more persistently and may predominate at moderate oxygen tensions [35,37,38,42]. These dynamics are further shaped by basal isoform expression, *EGLN*/PHD activity, protein and messenger ribonucleic acid (mRNA) stability, feedback regulation, and chromatin context. Rather than representing absolute rules, these patterns provide a useful conceptual framework for understanding why HIF-1α often dominates acute cellular adaptation, whereas HIF-2α more frequently orchestrates sustained tissue remodeling and intercellular communication.

One of the major advances in HIF biology has been the realization that oxygen sensing is fundamentally cell-type specific. Genome-wide studies demonstrate that HIF-1α and HIF-2α bind overlapping but distinct genomic regions, yet DNA binding alone is insufficient to predict transcriptional responses because chromatin accessibility, enhancer activity, cofactor availability, and cell lineage determine which genes are ultimately activated [39,40,41]. EPO regulation illustrates this concept particularly well. Although discovery of the HIF pathway stemmed from studies of oxygen-regulated EPO expression, later in vivo work revealed that HIF-2α is the principal regulator of EPO production in the kidney and liver, highlighting how individual physiological functions can become dominated by one HIF isoform despite extensive overlap in pathway architecture [43,44,45]. Thus, oxygen concentration alone does not determine HIF biology; cellular identity helps define the transcriptional program that hypoxia can access.

Additional regulatory layers further refine HIF-2α activity (Figure 2). Long noncoding ribonucleic acids (lncRNAs), RNA-binding proteins, chromatin regulators, inflammatory signals, and metabolic feedback can alter the amplitude, duration, and cell specificity of HIF responses. For example, the endothelial-enriched lncRNA *GATA2-AS1* modulates the balance between HIF-1α and HIF-2α signaling during acute hypoxia [42], whereas in pulmonary arterial hypertension HIF-2α induces *KMT2E-AS1* to promote pathogenic endothelial chromatin remodeling through stabilization of KMT2E [21]. Together, these examples illustrate that HIF-2α activity may be manipulated through cell-specific regulatory circuits in addition to direct pharmacological inhibition. These examples extend the concept of cell-specific HIF output from chromatin state to regulatory circuitry, suggesting that HIF-2α activity may be tuned indirectly through disease- and cell-enriched pathways rather than only through direct systemic inhibition or broad HIF stabilization.

For the lung, the practical implication is that HIF activity must be interpreted anatomically and temporally. Airway epithelial cells, alveolar epithelial cells, pulmonary endothelial cells, smooth muscle cells, pericytes, fibroblasts, and alveolar macrophages differ in oxygen exposure, basal *EPAS1* expression, chromatin state, inflammatory signaling, metabolic program, and developmental history. The remainder of this review therefore considers HIF-2α not simply as the chronic counterpart of HIF-1α, but in relation to cell identity, oxygen dose, signaling duration, and the regulatory circuits that determine whether hypoxic adaptation resolves injury or becomes maladaptive remodeling.

## 3. Human Genetics and Chronic Hypoxia Adaptation

Human high-altitude adaptation provides one of the clearest human examples that *EPAS1*/HIF-2α biology can be tuned without abolishing pathway function. Populations living for generations at high altitude have adapted to chronic hypobaric hypoxia through genetic changes that influence oxygen transport, vascular physiology, and systemic hypoxia tolerance [26,27,28]. These studies are important for pulmonary biology because they show that HIF pathway output is not simply “on” or “off,” but quantitatively shaped by regulatory and upstream oxygen-sensing variation. This principle provides a useful framework for therapeutic translation: in lung disease, the goal may often be to adjust the magnitude, location, or timing of HIF-2α activity rather than to eliminate or amplify the pathway globally.

The Tibetan high-altitude adaptation story remains the best characterized example. Genome-wide studies have identified strong signals of positive selection near *EPAS1* in Tibetan populations, with selected variants associated with lower hemoglobin concentration at altitude compared with non-adapted populations [26,27,28]. This phenotype is biologically important because excessive erythrocytosis can increase blood viscosity and contribute to maladaptive high-altitude physiology, whereas Tibetan highlanders tolerate chronic hypobaric hypoxia without the same degree of hemoglobin elevation. Physiological studies describing distinctive nitric oxide and vascular biology in Tibetan highlanders further reinforce that chronic hypoxia adaptation involves systemic oxygen-delivery programs rather than hemoglobin concentration alone [46,47].

A notable feature of the Tibetan *EPAS1* signal is that it lies largely in noncoding regulatory regions rather than in an obvious protein-altering mutation. The selected haplotype has a genetic structure best explained by introgression from Denisovan or Denisovan-related ancestry, making it a striking example of archaic introgression contributing to modern human adaptation [48]. Functional studies have begun to connect this genetic signal to HIF-2α pathway output: Tibetan-enriched *EPAS1* variation is associated with reduced *EPAS1* expression in human endothelial cells and placenta, while high-altitude alleles alter the activity of multiple *EPAS1* enhancers in cell-based assays, including cell-type- and oxygen-dependent effects [49,50]. These findings move the high-altitude story beyond association genetics and support a regulatory model in which noncoding variation tunes *EPAS1*/HIF-2α dosage.

The *EPAS1* story is also linked to *EGLN1*, which encodes PHD2, a major oxygen-dependent negative regulator of HIF-α stability (Figure 2). Tibetan populations show evidence of selection at both *EPAS1* and *EGLN1*, suggesting that long-term adaptation involves coordinated tuning of HIF-α abundance and upstream oxygen sensing rather than *EPAS1* variation alone [28,51,52,53]. Importantly, this biology is not reducible to “more HIF” or “less HIF.” Tibetan PHD2 variants have been reported to alter experimental PHD2 function and influence ventilatory responses to hypoxia, indicating that adaptation may reshape pathway output across tissues rather than uniformly suppressing or activating HIF signaling [54].

Andean highlanders illustrate a different route to altered HIF-2α biology. In contrast to the Tibetan signal, which is dominated by noncoding regulatory variation, studies in Andean populations have identified a coding *EPAS1* variant, rs570553380, encoding the HIF-2α H194R substitution. Selection at *EPAS1* and *GPR126* was identified in Andean high-altitude populations, and the HIF-2α H194R allele was later characterized as a hypomorphic variant with evidence of positive selection [55,56]. This variant is associated with relatively lower hematocrit in Andean highlanders and attenuates canonical HIF-2α target-gene expression and HIF-2α-ARNT heterodimerization in experimental systems [56,57]. The contrast between Tibetan and Andean adaptation is conceptually important: distinct genetic solutions appear to converge on altered HIF-2α output as a route to improved chronic hypoxia tolerance (Table 1) (Tables S3 and S4).

These high-altitude examples raise a broader question for pulmonary and critical-care biology. The Denisovan-derived Tibetan haplotype is unusual, but genetic variation across HIF-pathway coding and regulatory loci—including *EPAS1*, *EGLN1*, *VHL*, *ARNT*, enhancers, and noncoding RNA regions—is widespread in human populations [58]. Whether common or rare variants in these regions contribute to interindividual differences in hypoxia tolerance, pulmonary vascular responses, erythropoietic reserve, inflammatory remodeling, or recovery from acute lung injury remains largely unresolved. This represents an important frontier, particularly for noncoding regulatory elements and long noncoding RNAs that may tune HIF-2α activity in a cell-type-specific manner.

The implication for lung disease is indirect but important. High-altitude genetics does not predict the effect of HIF-2α modulation in pulmonary hypertension, fibrosis, emphysema, or acute lung injury. It does, however, establish three principles that are central to therapeutic translation. First, *EPAS1*/HIF-2α activity is important enough to be repeatedly detected by human population genetics under chronic hypoxic selection. Second, adaptive phenotypes appear to result from quantitative tuning of pathway output rather than complete pathway elimination. Third, many informative variants act through regulatory DNA, enhancer activity, or upstream oxygen-sensing components, indicating that HIF-2α biology can be modulated indirectly. Together, these principles support a model in which future HIF-2α-directed therapy in lung disease should be guided by dosage, cell type, disease phase, and the specific HIF-2α-dependent program being targeted.

## 4. HIF-2α in Lung Development and Cell-State Biology

**Figure 4:**
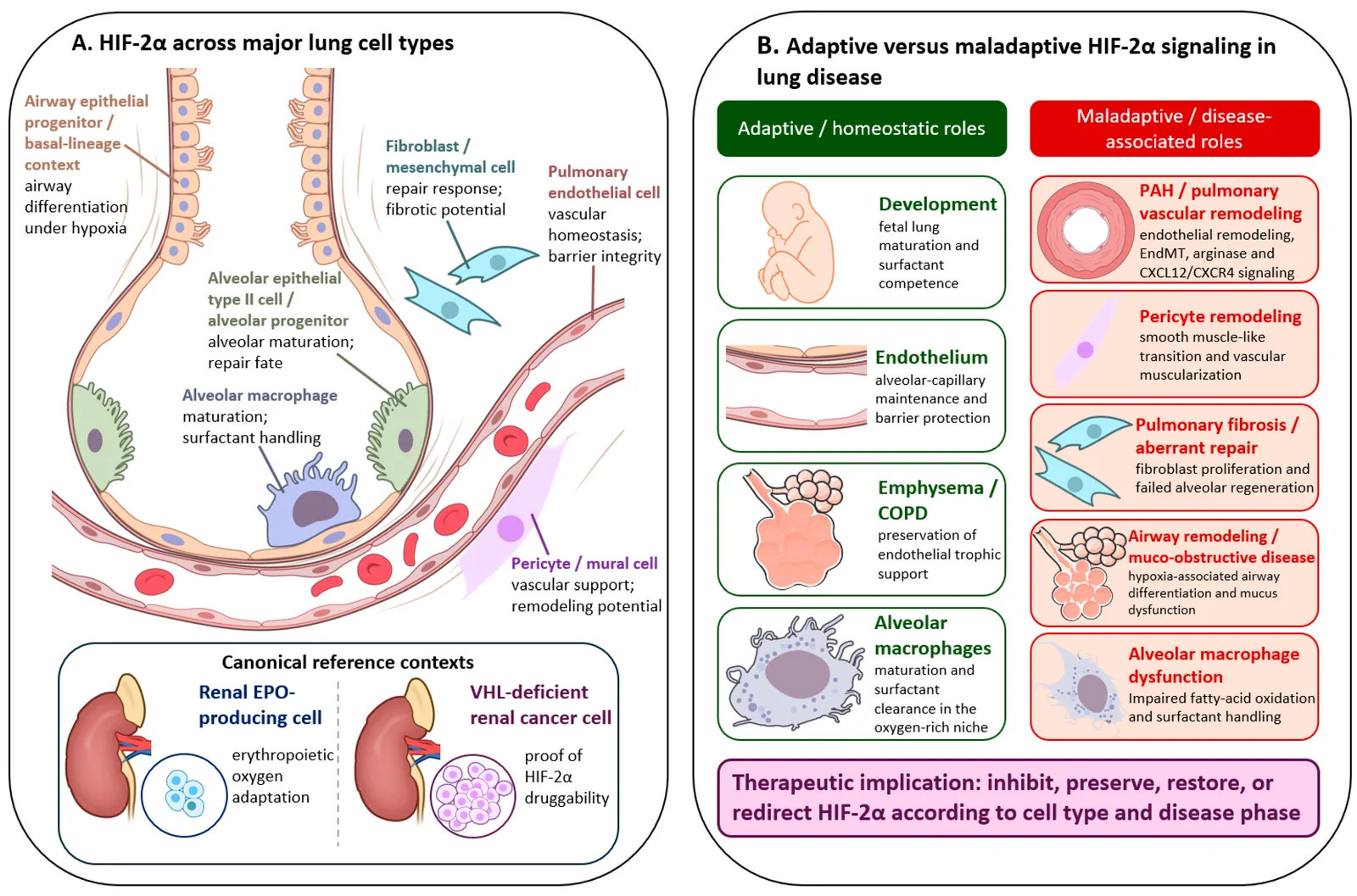
Context-dependent HIF-2α across lung cell types and disease states. (**A**) Schematic overview of the major lung cell types in which HIF-2α biology is relevant. In the developing and adult lung, HIF-2α contributes to endothelial homeostasis and barrier integrity, alveolar epithelial maturation and repair fate, airway differentiation under hypoxic stress, mesenchymal repair responses, and alveolar macrophage maturation and surfactant handling. Canonical non-lung reference contexts include renal EPO-producing cells, in which HIF-2α regulates erythropoietic oxygen adaptation, and VHL-deficient renal cancer, which established HIF-2α as a druggable transcription factor. (**B**) HIF-2α exerts adaptive or maladaptive effects depending on cell type and disease context. Adaptive roles include fetal lung maturation, maintenance of the alveolar–capillary unit, endothelial barrier protection, and support of alveolar macrophage maturation in the oxygen-rich alveolar niche. Maladaptive or persistent HIF-2α signaling has been implicated in pulmonary vascular remodeling and pulmonary arterial hypertension, pericyte transition toward smooth muscle-like states, fibroblast proliferation and aberrant epithelial repair in fibrosis, airway remodeling under chronic hypoxic stress, and impaired surfactant handling in alveolar macrophages. The figure highlights the central translational principle of this review: therapeutic strategies should aim to inhibit, preserve, restore, or redirect HIF-2α according to cell type, disease phase, and biological endpoint.

The lung is a compelling organ in which to study HIF-2α because its development requires one of the most dramatic oxygen transitions in mammalian life: the shift from a relatively hypoxic, liquid-filled fetal organ to an air-exposed tissue responsible for gas exchange (Figure 4). This transition requires coordinated epithelial maturation, surfactant production, vascular perfusion, alveolar surface stability, immune readiness, and epithelial–endothelial coupling. Hypoxia-inducible factor (HIF) signaling is positioned at the center of this process because it links oxygen tension to growth-factor expression, epithelial differentiation, endothelial maturation, and metabolic state.

Genetic models first established that HIF-2α is required for normal fetal lung maturation. Loss of HIF-2α in mice causes fatal neonatal respiratory distress associated with impaired surfactant production and defective type II pneumocyte maturation, while perturbation of vascular endothelial growth factor (VEGF) signaling phenocopies aspects of this respiratory failure [59,60]. These findings placed HIF-2α upstream of a coordinated program linking oxygen sensing, VEGF-dependent epithelial–vascular communication, surfactant competence, and alveolar–capillary maturation. Subsequent studies reinforced the broader importance of HIF signaling in immature lung development and bronchopulmonary dysplasia models, including evidence that HIF stabilization can preserve lung growth and structure in experimental neonatal lung injury [61,62,63]. These data establish an important principle for the rest of this review: HIF-2α is not intrinsically pathological, and in the appropriate developmental context it supports lung maturation and epithelial–vascular coordination.

Recent human organoid studies extend this developmental principle into epithelial cell-state biology. In human lung progenitor systems, hypoxia promotes airway differentiation and limits alveolar differentiation through HIF-dependent mechanisms. HIF-2α appears to bias progenitor fate toward basal cell programs while restraining secretory, neuroendocrine, and alveolar gene-expression programs. In these models, genetic knockdown of *EPAS1* or pharmacologic HIF-2α inhibition under hypoxia reduced basal cell marker expression and increased markers of alternative epithelial lineages, whereas HIF-2α stabilization under normoxic conditions promoted basal cell differentiation and suppressed non-basal lineage markers [64]. These data position HIF-2α as a regulator of epithelial fate choice rather than simply a downstream marker of hypoxic exposure.

A related pattern has been observed in more mature alveolar epithelial systems. In alveolar type II (AT2) cell-derived organoids, chronic hypoxia can reduce AT2 identity and promote airway-like differentiation, including basal-like programs that persist after reoxygenation in some experimental conditions [64]. This observation provides a conceptual bridge between development and adult repair. During normal maturation, oxygen-dependent signaling helps coordinate epithelial lineage allocation; after injury, persistent or ectopic activation of similar programs may redirect alveolar progenitors away from effective regeneration and toward airway metaplasia or aberrant repair. HIF-2α is unlikely to explain epithelial remodeling alone, but these data establish oxygen tension and HIF isoform activity as plausible upstream regulators of epithelial plasticity after lung injury.

HIF-2α also intersects with mesenchymal and vascular maturation. Lung development depends on reciprocal signaling among epithelium, endothelium, and mesenchyme, and HIF-2α is especially relevant to this multicellular architecture because of its enrichment in endothelial and stromal compartments. Its regulation of VEGF-dependent signaling, endothelial differentiation, and vascular morphogenesis helps explain why HIF-2α perturbation can affect both epithelial surfactant competence and vascular development [10,60]. This principle remains relevant in adult disease, where failed repair often reflects combined disruption of epithelial integrity, endothelial homeostasis, and mesenchymal restraint rather than dysfunction of a single compartment.

Alveolar macrophage maturation adds an immune-cell dimension to lung HIF biology. Unlike many tissue macrophage populations, alveolar macrophages mature after birth in an oxygen-rich niche and acquire specialized functions in surfactant handling, lipid metabolism, host defense, and maintenance of alveolar homeostasis. Recent work using *Vhl*-deficient mouse alveolar macrophage models indicates that persistent HIF activity impairs terminal maturation and self-renewal, with HIF-1α and HIF-2α contributing overlapping but non-identical effects. HIF-1α preferentially promotes glycolytic reprogramming, whereas HIF-2α disrupts fatty-acid oxidation and surfactant clearance, including in experimental pulmonary alveolar proteinosis models [25]. This finding shows that postnatal alveolar homeostasis requires not only activation of oxygen-sensing pathways during development, but also restraint of inappropriate HIF activity after adaptation to air breathing.

Collectively, these studies establish HIF-2α as more than a hypoxia-responsive transcription factor. They position HIF-2α as a context-dependent regulator of lung maturation, epithelial–vascular communication, immune specialization, and repair-state plasticity. This perspective provides an important framework for interpreting adult lung disease, where reactivation of developmental or stress-response programs may either restore tissue homeostasis or drive maladaptive remodeling depending on the compartment and timing of pathway activation.

## 5. HIF-2α in Non-Cancer Lung Disease

In adult lung disease, HIF-2α programs that support development and homeostasis may become protective, maladaptive, or mixed depending on the injured compartment and disease phase. This is the central translational challenge. In pulmonary vascular disease, fibrosis, emphysema, acute lung injury, and airway or macrophage-associated disorders, HIF-2α may either amplify pathological remodeling or preserve essential homeostatic functions. The following sections therefore consider HIF-2α not as a generic marker of hypoxia, but as a disease- and compartment-specific regulator of lung injury, repair, and remodeling (Figure 4).

### 5.1. Pulmonary Vascular Remodeling and Pulmonary Arterial Hypertension

Pulmonary arterial hypertension (PAH) is the non-cancer lung disease in which the case for pathogenic HIF-2α activity is currently most developed. PAH is characterized by progressive pulmonary vascular remodeling, increased pulmonary vascular resistance, right ventricular overload, and premature mortality. This connection to oxygen sensing is reinforced by the preclinical literature, where chronic hypoxia and SU5416/hypoxia models remain among the most widely used experimental platforms for studying pulmonary vascular remodeling, alongside monocrotaline and other injury-based approaches [65,66]. Its vascular pathology is multicellular: endothelial dysfunction, smooth muscle proliferation, pericyte expansion or transition, fibroblast activation, inflammatory recruitment, altered metabolism, and impaired vascular tone all contribute to disease progression. This architecture makes PAH a natural setting for HIF-2α biology because HIF-2α is enriched in endothelial and vascular-associated compartments, and regulates programs linking oxygen sensing to vascular remodeling, arginine metabolism, chemokine signaling, and cell-state transition [10,35,67].

The endothelial compartment provides the most direct mechanistic entry point. In experimental hypoxic pulmonary hypertension, pulmonary endothelial HIF-2α is required for the full increase in right ventricular systolic pressure and vascular remodeling. This effect is mediated in part through an endothelial HIF-2α–arginase axis, in which HIF-2α induces arginase expression, alters L-arginine availability, and disrupts nitric oxide homeostasis, thereby connecting chronic hypoxia, endothelial metabolism, and pulmonary vascular tone [19]. Human and experimental data also show increased HIF-2α in lung vascular endothelial cells in severe pulmonary hypertension, where HIF-2α promotes SNAI1/2 expression, endothelial-to-mesenchymal transition (EndMT), vascular occlusion, and severe pulmonary hypertensive remodeling [68]. Together, these studies position endothelial HIF-2α as an active driver of pulmonary vascular remodeling rather than a passive marker of hypoxic stress.

HIF-2α also intersects with established PAH pathways. EndMT has been linked to altered bone morphogenetic protein receptor type 2 (BMPR2) signaling and occlusive vascular remodeling in PAH, providing a bridge between canonical PAH genetics, endothelial plasticity, and HIF-driven transcriptional programs [69]. In parallel, endothelial loss or dysfunction of *EGLN1*/PHD2, a major oxygen-dependent negative regulator of HIF-α stability, can produce severe pulmonary vascular remodeling through HIF-2α-dependent mechanisms, including C-X-C motif chemokine ligand 12 (CXCL12) signaling [70]. These findings connect the biochemical oxygen-sensing machinery directly to remodeling-prone endothelial states, even when the initiating event is not simply low environmental oxygen.

More recent studies have expanded the HIF-2α model from endothelial activation to epigenetic and noncoding RNA amplification. A PAH-associated risk allele, rs73184087, has been linked to allele-specific HIF-2α binding and induction of the endothelial long noncoding ribonucleic acid (lncRNA) *KMT2E-AS1*. *KMT2E-AS1* stabilizes KMT2E and increases histone 3 lysine 4 trimethylation (H3K4me3)-associated pathogenic endothelial activity, creating a positive-feedback circuit in which hypoxia, genetic susceptibility, noncoding RNA regulation, chromatin remodeling, and HIF-2α signaling reinforce one another [21]. This mechanism suggests that HIF-2α-dependent vascular remodeling may be sustained through cell-specific regulatory circuits, not only through direct HIF-2α transcriptional activity.

The vascular wall contribution is not endothelial-only. Pericytes occupy a strategic position at the interface between endothelial cells and the mural compartment, and recent work identifies them as HIF-2α-responsive contributors to pulmonary vascular remodeling. In human PAH samples and experimental systems, HIF-2α-high pericytes show transcriptomic and functional features of transition toward smooth muscle-like contractile cells. HIF-2α overexpression in human lung pericytes increases contractility and disrupts endothelial–pericyte tube formation, while pericyte-dominant HIF-2α activation in mice promotes vascular muscularization, pulmonary hypertension, right ventricular hypertrophy, and vascular leakage. Pharmacologic antagonism of C-X-C chemokine receptor 4 (CXCR4) attenuates aspects of this phenotype, linking pericyte HIF-2α activity to CXCL12/CXCR4-dependent remodeling programs [20]. These findings strengthen the view that HIF-2α functions as a multicompartment remodeling signal in PAH rather than a pathway confined to endothelial cells.

Smooth muscle and inflammatory compartments likely broaden this model, although the HIF-2α-specific evidence is less developed than in the endothelial and pericyte literature. Hypoxia-dependent pulmonary artery smooth muscle cell proliferation, altered mitochondrial metabolism, calcium handling, glycolytic reprogramming, apoptosis resistance, and inflammatory remodeling have all been implicated in pulmonary vascular disease, with HIF signaling positioned upstream of several of these remodeling-associated phenotypes [71,72,73]. Immune-cell HIF-2α programs may also contribute through arginase-dependent effects on nitric oxide biology and tissue remodeling, consistent with evidence that macrophage HIF-1α and HIF-2α drive distinct inflammatory, metabolic, and migratory programs [19,74,75]. Together, these mechanisms suggest that HIF-2α biology operates within a broader vascular niche shaped by hypoxia, inflammation, metabolism, and cell-state plasticity.

The therapeutic implications are unusually direct for a non-cancer HIF-2α use case. Genetic deletion or knockdown of HIF-2α attenuates pulmonary vascular remodeling in experimental systems, and selective pharmacologic HIF-2α antagonism has been reported to reduce right ventricular systolic pressure, vascular remodeling, right ventricular hypertrophy, and right heart failure in rodent models of pulmonary hypertension [76,77,78]. Notably, belzutifan (MK-6482) also reduced pulmonary hypertension in genetically driven mouse models of constitutive HIF-2α activation, including *VhlR200W* and *Irp1*-deficient mice, providing complementary evidence that pharmacological HIF-2α inhibition can reverse pulmonary vascular dysfunction arising from dysregulated oxygen sensing [79]. These studies support the idea that HIF-2α is not only associated with PAH pathobiology but is functionally targetable. However, the same multicompartment biology that makes HIF-2α attractive also complicates clinical translation: inhibition of HIF-2α-active endothelial, pericyte, or smooth muscle remodeling states may be beneficial, whereas indiscriminate suppression could affect endothelial programs required for barrier function or alveolar homeostasis.

PAH therefore provides the clearest current proof-of-concept for HIF-2α as a therapeutically actionable remodeling node in non-cancer lung disease. HIF-2α-associated mechanisms connect chronic hypoxia and impaired oxygen sensing with endothelial metabolism, EndMT, CXCL12/CXCR4 signaling, lncRNA-mediated chromatin remodeling, and pericyte transition within a broader inflammatory and metabolic vascular disease program. At the same time, PAH illustrates why future clinical strategies will need to identify the HIF-2α-active compartments driving disease rather than simply suppressing HIF-2α throughout the lung.

### 5.2. Pulmonary Fibrosis and Aberrant Repair

Unlike pulmonary arterial hypertension, where HIF-2α has been linked to a broad vascular remodeling program, pulmonary fibrosis raises a different question: can persistent HIF-2α activity redirect epithelial and mesenchymal repair toward maladaptive cell states? Although much of the pulmonary fibrosis literature has focused on HIF-1α, or combined HIF signaling, emerging studies using isoform-specific genetic and pharmacological perturbations have identified distinct contributions of HIF-2α to fibroblast proliferation and aberrant epithelial repair. These findings are supported by mechanistic studies in human pulmonary fibroblasts, human epithelial organoid models, and conditional genetic experiments in mice, although their relevance to established human pulmonary fibrosis remains incompletely defined.

Human idiopathic pulmonary fibrosis (IPF) tissue provides initial support for altered HIF pathway balance. Increased expression of HIF-1α and HIF-2α, together with reduced HIF-3α, has been reported in IPF lung tissue and IPF-derived fibroblasts, with HIF-α expression correlating with the myofibroblast marker α-smooth muscle actin [80]. These findings do not prove that HIF-2α drives fibrosis, but they support the view that the hypoxic response in IPF is dysregulated rather than simply adaptive. This distinction matters because the fibrotic lung contains heterogeneous microenvironments, including poorly ventilated regions, remodeled vasculature, dense matrix, inflammatory niches, and injured epithelium, each of which may generate distinct HIF outputs.

Mechanistic studies in human fibroblasts provide more direct evidence for HIF-2α-specific activity. In adult human IPF lung fibroblasts, hypoxia induces *microRNA-210* (*miR-210*) through HIF-2α, and *miR-210* promotes fibroblast proliferation in part by repressing MAX network transcriptional repressor (MNT), a c-Myc inhibitor [81]. In that system, inhibition of HIF-2α, rather than HIF-1α, reduced the hypoxia-induced proliferative response. A second line of evidence links hypoxia-induced proliferation in primary human pulmonary fibroblasts to HIF-2α-dependent activation of nuclear factor of activated T cells c2 (NFATc2), supporting a mechanism in which HIF-2α contributes to mesenchymal expansion rather than acting only as a marker of low oxygen tension [82]. Together, these studies suggest that HIF-2α can support fibroblast persistence through proliferative and transcriptional circuits that are at least partly distinct from classical HIF-1α programs. Importantly, both studies used isoform-specific perturbations to distinguish HIF-2α from HIF-1α activity, although their findings primarily establish mechanisms in cultured human pulmonary fibroblasts rather than a causal role in the progression of human pulmonary fibrosis.

Recent work provides stronger isoform-specific evidence linking HIF-2α to aberrant epithelial repair. In human IPF lungs, HIF-2α activity was associated with aberrant epithelial populations [23]. In in vivo mouse models of repetitive lung injury, genetic or pharmacologic HIF-2α inhibition reduced aberrant epithelial states, improved alveolar epithelial repair, and attenuated fibrotic remodeling [23]. Consistent effects were observed in both human and mouse organoids, in which HIF-2α inhibition promoted alveolar epithelial differentiation and repair [23]. These findings are consistent with human lung organoid data showing that chronic hypoxia can redirect alveolar type II (AT2)-derived cells toward airway-like states, with HIF-2α-specific perturbation associated with a bias of lung progenitors toward basal cell programs while restraining alveolar differentiation [64]. Together, these studies suggest that HIF-2α may contribute to fibrosis not only through fibroblasts, but also by altering the epithelial repair trajectories that determine whether injury resolves or progresses toward remodeling.

This epithelial framing helps reconcile the apparently paradoxical roles of HIF-2α across lung biology. During development, HIF-2α supports fetal lung maturation, surfactant competence, and epithelial–vascular coupling; after repetitive adult injury, persistent or ectopic HIF-2α activation may redirect epithelial cells toward maladaptive repair states. Similarly, hypoxia-driven fibroblast proliferation may be useful during controlled wound repair but harmful when it persists in a stiff, inflamed, matrix-rich lung.

Rather than establishing HIF-2α as the dominant regulator of pulmonary fibrosis, current evidence positions it as an emerging determinant of persistent epithelial and fibroblast states that prevent successful regeneration. Defining these HIF-2α-dependent repair-state trajectories may ultimately prove more informative than measuring pathway activation alone.

### 5.3. COPD, Emphysema, and Endothelial Homeostasis

Chronic obstructive pulmonary disease (COPD) and emphysema provide an important counterpoint to pulmonary hypertension and fibrosis because they illustrate a setting in which loss of HIF-2α activity may be pathogenic. Emphysema is characterized by alveolar destruction, distal airspace enlargement, microvascular rarefaction, impaired gas exchange, and defective repair. Although COPD is often framed primarily as an airway and inflammatory disease, the emphysema phenotype also reflects failure of the alveolar–capillary unit, including vascular dysfunction and loss of endothelial trophic support. In this context, endothelial HIF-2α appears less like a maladaptive remodeling signal and more like a homeostatic program required to maintain distal lung structure.

Human and experimental data support this model. In emphysematous human lung tissue, pulmonary endothelial HIF-2α expression is reduced, and endothelial loss of HIF-2α in mice produces an emphysema-like phenotype characterized by alveolar enlargement, microvascular loss, impaired angiocrine support, and reduced hepatocyte growth factor (HGF) signaling [22]. Earlier work also showed that endothelial HIF-2α is required for airway microvascular integrity and that disruption of endothelial HIF-2α can contribute to airway inflammation, remodeling, and lung-function impairment [83]. Together, these studies suggest that endothelial HIF-2α helps maintain the epithelial–endothelial niche required for distal lung structure and repair.

This interpretation has direct therapeutic implications. In pulmonary arterial hypertension and selected fibrotic repair states, persistent HIF-2α activity in remodeling-prone vascular, epithelial, fibroblast, or pericyte compartments may amplify disease. In emphysema, by contrast, insufficient endothelial HIF-2α activity may remove an adaptive program required for microvascular maintenance and alveolar homeostasis. A recent COPD endotyping framework has therefore proposed HIF-2α as a potential molecular switch between vascular-predominant and airway-predominant COPD biology, with reduced endothelial HIF-2α linked particularly to emphysema-relevant vascular loss [84].

The COPD literature also intersects with macrophage and airway epithelial HIF biology, although HIF-2α-specific evidence in these compartments is less mature. Persistent HIF-2α activity can impair fatty-acid oxidation and surfactant clearance in experimental alveolar macrophage models, while epithelial hypoxia/HIF signaling can influence mucus production, ion transport, barrier function, and airway remodeling, often through HIF-1α or combined HIF activity rather than HIF-2α alone [25,85,86]. These observations broaden the COPD framework but do not replace the key HIF-2α point: emphysema is currently the clearest example in which preserving or restoring endothelial HIF-2α may be more rational than inhibiting it.

COPD and emphysema therefore provide a critical counterweight to the inhibition-focused logic that emerges from pulmonary vascular remodeling and selected fibrotic repair states. In emphysema-predominant disease, the relevant therapeutic question may not be how to suppress HIF-2α, but how to preserve or restore endothelial HIF-2α programs that maintain the alveolar–capillary unit. This distinction makes COPD an important test case for determining whether HIF-2α-directed strategies can be tailored to pathological activation, protective loss, or spatially mixed pathway activity.

### 5.4. Mixed Remodeling Phenotypes: IPF-PH, CPFE, and COPD-PH

Clinically overlapping phenotypes such as pulmonary hypertension associated with idiopathic pulmonary fibrosis (IPF-PH), combined pulmonary fibrosis and emphysema (CPFE), and chronic obstructive pulmonary disease-associated pulmonary hypertension (COPD-PH) illustrate why HIF-2α cannot be approached as a uniform therapeutic target. These syndromes combine compartments in which HIF-2α may have opposing implications: pulmonary vascular remodeling and fibrotic repair may involve persistent or maladaptive HIF-2α activation, whereas emphysema may reflect loss of endothelial HIF-2α activity required for microvascular maintenance and alveolar homeostasis [22,23,68]. CPFE is particularly instructive because fibrotic remodeling, emphysematous vascular rarefaction, gas-exchange impairment, and pulmonary vascular disease can coexist within the same clinical syndrome [87].

These mixed phenotypes argue for a compartment-aware approach to HIF-2α modulation. In fibrosis with severe pulmonary vascular remodeling, HIF-2α inhibition could be biologically attractive if HIF-2α-active endothelial, pericyte, epithelial, or fibroblast states are driving disease progression. In emphysema-predominant disease, however, indiscriminate HIF-2α inhibition could plausibly worsen endothelial rarefaction or impair epithelial–endothelial trophic support. COPD-PH and CPFE may therefore require biomarkers that distinguish pathogenic HIF-2α activation in remodeling vascular or fibrotic niches from protective HIF-2α activity in residual alveolar–capillary units. Spatial profiling, single-cell analysis, radiographic phenotyping, and disease-trajectory biomarkers may be necessary before HIF-2α-directed therapy can be rationally tested in these overlapping syndromes. In practice, candidate stratification would likely need to consider the dominant structural phenotype (e.g., fibrosis vs. emphysema), disease stage, and the trajectory of the pulmonary vascular remodeling. In future, biomarkers of HIF-2α activity may guide the appropriate magnitude and direction of HIF-2α modulation.

### 5.5. ALI/ARDS and Endothelial Barrier Biology

Acute lung injury (ALI) and acute respiratory distress syndrome (ARDS) illustrate why the timing of HIF-2α activity may be as important as the cell type in which it occurs. In pulmonary vascular remodeling, sustained endothelial HIF-2α can contribute to pathological remodeling; in acute inflammatory injury, endothelial HIF-2α may instead preserve vascular barrier integrity and limit edema formation. ARDS is characterized by disruption of the alveolar–capillary barrier, inflammatory cell recruitment, protein-rich alveolar edema, impaired lung compliance, and severe gas-exchange failure. In the international LUNG SAFE study, ARDS accounted for 10.4% of intensive care unit admissions and 23.4% of mechanically ventilated patients, underscoring its clinical importance as a syndrome of acute barrier failure and impaired repair [88].

Experimental studies suggest that endothelial HIF-2α can stabilize the pulmonary vascular barrier during acute injury. In models of inflammatory lung injury, endothelial HIF-2α activation preserved adherens junction integrity through induction of vascular endothelial protein tyrosine phosphatase (VE-PTP), promoting vascular endothelial cadherin (VE-cadherin) dephosphorylation, limiting VE-cadherin internalization, and reducing vascular leak [24,89]. This places HIF-2α within an acute endothelial barrier-protective program, rather than the chronic remodeling program emphasized in pulmonary vascular disease.

The repair phase appears to involve different HIF isoform biology. After polymicrobial sepsis or endotoxemia in a mouse model, endothelial HIF-1α, rather than HIF-2α, is rapidly induced in lung vascular endothelial cells and supports endothelial proliferation, vascular repair, inflammation resolution, and survival through a forkhead box M1 (FoxM1)-dependent program [90]. Thus, acute lung injury provides an example in which HIF-2α may help preserve barrier integrity during the injury phase, while HIF-1α may contribute more strongly to endothelial regeneration during recovery.

This distinction is important for therapeutic interpretation. HIF-2α inhibition that may be attractive in pulmonary vascular remodeling could be harmful if applied during a phase when endothelial HIF-2α is maintaining junctional integrity and limiting edema. Conversely, broad HIF stabilization could support selected barrier or repair programs while amplifying maladaptive responses in other compartments. ALI/ARDS therefore reinforces that HIF-2α modulation in lung disease should be matched to disease phase and cellular compartment, not simply to the presence of hypoxia.

### 5.6. Airway Epithelial Differentiation and Muco-Obstructive Disease

Airway epithelial disease provides a relevant but more limited extension of HIF-2α biology. Human lung organoid studies suggest that hypoxia and HIF signaling can bias lung progenitors toward airway differentiation while restraining alveolar fate. Isoform-specific perturbation implicates HIF-2α in promoting basal-lineage programs and restraining alveolar differentiation [64]. These findings support the idea that regional hypoxia could reinforce epithelial state changes after airway obstruction, infection, or repetitive injury, although the disease-specific role of HIF-2α in airway remodeling remains incompletely defined.

However, most mucus-focused data implicate HIF-1α or combined HIF activity more clearly than HIF-2α alone. HIF signaling has been linked to goblet-cell hyperplasia, mucin induction, impaired mucociliary function, epithelial barrier dysfunction, and altered airway surface hydration [85,91]. In muco-obstructive models, epithelial hypoxia can increase mucin 5B (MUC5B) and enhance epithelial sodium channel (ENaC)-dependent fluid absorption through HIF-1α/HIF-2α-dependent mechanisms, worsening mucus hyperconcentration and airway injury [86]. Airway disease is therefore best viewed as HIF-relevant but not yet HIF-2α-specific; therapeutic interpretation will require clearer separation of HIF-2α-dependent effects from broader hypoxia/HIF activation.

### 5.7. Alveolar Macrophage Maturation and Surfactant Handling

Alveolar macrophages provide a lung-resident immune-cell example of HIF-2α niche dependence. Unlike many hypoxia-responsive inflammatory cells, mature alveolar macrophages occupy an oxygen-rich environment and are specialized for surfactant clearance, lipid metabolism, host defense, and maintenance of alveolar homeostasis. Recent work using Vhl-deficient alveolar macrophage mouse models showed that persistent HIF activity impairs terminal maturation and self-renewal, with HIF-1α and HIF-2α regulating overlapping but distinct programs. HIF-1α preferentially promoted glycolytic reprogramming, whereas HIF-2α disrupted fatty-acid oxidation and surfactant clearance, including in experimental pulmonary alveolar proteinosis models [25].

This finding broadens the lung HIF-2α framework beyond structural remodeling. It suggests that inappropriate HIF-2α activation in the alveolar immune niche could impair surfactant handling and alveolar stability, particularly in settings where edema, atelectasis, infection, or inflammation create regional hypoxic microenvironments. Direct disease-specific evidence remains limited, but the concept is important: in a cell type specialized for life in an oxygen-rich airspace, persistent activation of hypoxia-response programs may disrupt rather than restore homeostasis. The principal HIF-2α-relevant cell types, functional effects, levels of evidence, and potential therapeutic implications across these pulmonary disease states are summarized in Table S5.

## 6. Therapeutic Considerations: Adverse Effects, Strategies for Mitigation and Targeted Delivery, and Roadmap for Precision Modulation Rather than Global Activation or Inhibition

The clinical success of belzutifan demonstrates that HIF-2α is a tractable therapeutic target. Selective HIF-2α inhibition has validated this pathway in von Hippel–Lindau (VHL) disease-associated tumors and advanced clear cell renal cell carcinoma, establishing a drug model that is already familiar to clinicians and immediately relevant to pulmonary translation [15,16,17,92,93]. Importantly, this model should not be viewed as limited to oncology. In pulmonary arterial hypertension or selected fibrotic repair states, systemic or orally available HIF-2α inhibition may still be appropriate if the relevant disease biology is dominated by pathogenic HIF-2α-active vascular, epithelial, fibroblast, or pericyte compartments. Indeed, the preclinical pulmonary vascular literature already supports the idea that HIF-2α can be functionally targetable outside cancer. The central challenge is therefore not whether current-generation inhibitors are relevant to lung disease, but how to identify the patients, disease phases, and tissue compartments in which inhibition is likely to be beneficial rather than disruptive. In emphysema or acute endothelial barrier injury, for example, indiscriminate inhibition could plausibly interfere with endothelial programs required for alveolar–capillary maintenance or vascular integrity [22,24]. Thus, pulmonary HIF-2α therapeutics should build on the oncology drug paradigm while extending it toward disease-phase and compartment-aware use.

### 6.1. Adverse Effects with Belzutifan and Systemic Inhibition in Clinical Contexts

The most common reported adverse effects with belzutifan are anemia and hypoxia [93], both a consequence of on-target toxicity due to the central role of HIF-2α in erythropoietin production and other central processes. This is mitigated clinically by adjusting the dose, balancing efficacy of treatment against the observed on-target side effects. These on-target effects may be particularly relevant in patients with chronic respiratory disease, in whom baseline hypoxemia or limited erythropoietic reserve could reduce tolerance of systemic HIF-2α inhibition. In addition, because endothelial HIF-2α contributes to pulmonary microvascular and alveolar–capillary homeostasis in some disease contexts, systemic inhibition could theoretically disrupt protective vascular programs, particularly in emphysema-predominant or otherwise microvascular-compromised lungs.

### 6.2. Potential Strategies for Mitigating On-Target Toxicity from Systemic Inhibition

Given the systemic, on-target adverse effects of anemia and hypoxia, localized pulmonary delivery represents a potential strategy for reducing systemic exposure to HIF-2α inhibitors. Inhaled or aerosolized formulations could, in principle, increase drug exposure within the lung [11,94] while limiting inhibition of HIF-2α-dependent erythropoietin production in the kidney and liver, although pulmonary formulations of current HIF-2α inhibitors would still require dedicated pharmacokinetic, safety, and efficacy studies.

We have highlighted that HIF-2α can have different effects in the lung based on cell type (pulmonary endothelial cells, alveolar type II (AT2) cells, pericytes, lung fibroblasts/mesenchymal cells, alveolar macrophages, pulmonary artery smooth muscle cells) and disease state (PAH, IPF vs. emphysema, COPD). Consequently, more cell- and compartment-restricted delivery strategies may ultimately provide greater control over HIF-2α modulation in the lung [95]. Emerging pulmonary drug-delivery platforms include lipid nanoparticles and viral or nonviral gene-delivery systems, which can be engineered through particle properties, vector tropism, or cell-restricted regulatory elements to enrich delivery or expression in selected pulmonary compartments. Further development of these approaches may improve their ability to achieve sufficiently selective and durable targeting in diseased human lungs and will require further validation [96].

### 6.3. Roadmap for Precision Modulation

A practical translational roadmap should begin with biological stratification. Future studies should define HIF-2α-active cell states in human lung disease using spatial transcriptomics, single-cell multi-omics, chromatin profiling, organoid injury models, lineage tracing, and longitudinal sampling across disease phases. The key question is not only whether *EPAS1* or HIF-2α target genes are increased, but what that activity represents: adaptive repair, failed regeneration, vascular remodeling, epithelial metaplasia, macrophage dysfunction, or mesenchymal persistence—and the local oxygen, metabolic, and redox context in which it occurs. This distinction may be especially important in mixed phenotypes such as combined pulmonary fibrosis and emphysema (CPFE), chronic obstructive pulmonary disease-associated pulmonary hypertension (COPD-PH), or fibrotic interstitial lung disease with pulmonary hypertension, where protective and pathogenic HIF-2α programs may coexist in different regions of the same lung. Once the relevant HIF-2α-active state has been defined, therapeutic design can proceed along three complementary axes: whether to target HIF-2α directly or indirectly, how to deliver the intervention to the relevant lung compartment, and whether to modulate the entire pathway or a disease-specific downstream circuit.

Dosage sensitivity may not be uniform across pulmonary disorders. In PAH or selected fibrotic states, the therapeutic goal may be partial suppression of excessive HIF-2α activity, whereas in emphysema-predominant disease, preservation or restoration of endothelial HIF-2α activity may be preferable. One pathway forward may be the development of biomarkers of HIF-2α activity and disease trajectory that can guide the direction and magnitude of modulation.

The effects of HIF-2α-directed therapy may benefit from consideration of concomitant disease-specific treatment. In PAH and fibrotic lung disease, existing therapies may alter vascular, inflammatory, or fibrotic cell states that overlap with HIF-2α-regulated pathways. To our knowledge, clinically meaningful pharmacodynamic or drug–drug interactions have not yet been defined and should be assessed prospectively in preclinical and early-phase studies.

Therapeutic specificity could then be pursued by matching the molecular modality to the biological objective. When a pathogenic state is clearly HIF-2α-dependent, direct pharmacologic inhibition remains the most immediate strategy because it builds on an established clinical drug class. Where the goal is to reduce *EPAS1* expression or interrupt a disease-associated transcript, RNA-based approaches such as small interfering RNA (siRNA), antisense oligonucleotides, or other RNA-targeting strategies may offer a way to tune pathway components with greater sequence specificity [97,98,99,100]. Conversely, where HIF-2α activity appears insufficient, as may occur in endothelial compartments in emphysema-predominant disease, restoration strategies could theoretically include messenger RNA (mRNA), gene-regulatory, or gene-augmentation approaches, although these remain more speculative for HIF-2α lung biology than direct inhibition. Across these modalities, delivery will be a major determinant of feasibility, with inhaled administration, lung-tropic nanoparticles, antibody-guided delivery, and other targeted vehicles offering potential routes to improve compartment selectivity [99,101].

For gene delivery strategies, specificity may also be improved at the level of expression control. Promoter-restricted, enhancer-informed, or otherwise cell-selective regulatory designs could be used to narrow expression to relevant lung compartments, tune expression level, or reduce off-target transgene activity in non-target cells [102]. This distinction is important because delivery to the lung does not automatically equal delivery to the correct pulmonary cell state. A therapy that reaches the lung broadly may still need regulatory control to distinguish endothelial, epithelial, fibroblast, pericyte, macrophage, or airway compartments. In this sense, the emerging toolkit of targeted delivery and regulatory design could allow HIF-2α biology to be manipulated with greater spatial and quantitative control than systemic small-molecule therapy alone, while still building on the proof-of-principle established by current HIF-2α inhibitors.

A third level of specificity is pathway or circuit selection. When only part of the HIF-2α program is pathogenic, it may be preferable to target downstream mediators that carry disease-relevant outputs while preserving beneficial HIF-2α functions in other compartments. Candidate examples emerging from the biology reviewed above include CXCL12/CXCR4 signaling in vascular remodeling, arginase-dependent nitric oxide imbalance, microRNA-210 (miR-210)-associated fibroblast proliferation, and *KMT2E-AS1*/KMT2E-dependent endothelial chromatin remodeling [19,20,21,81]. Cell-enriched noncoding RNAs and regulatory elements may be especially useful in this regard because they offer access to HIF-pathway control with greater compartment specificity than systemic HIF-2α inhibition or broad prolyl hydroxylase domain enzyme inhibition. For example, *GATA2-AS1* has been linked to endothelial regulation of acute HIF-1α/HIF-2α balance, whereas *KMT2E-AS1* participates in HIF-2α-driven endothelial pathology in pulmonary hypertension [21,42].

The near-term goal should therefore be an investigation-to-therapy pipeline. First, define the HIF-2α-active cellular state in human tissue. Second, determine whether that state is adaptive, maladaptive, or mixed at the relevant disease phase. Third, identify whether the therapeutic objective is inhibition, preservation, restoration, or downstream circuit modulation. Finally, match that objective to a molecular strategy and delivery route capable of reaching the relevant lung compartment. This framework keeps HIF-2α therapeutics anchored to the druggable pathway established in oncology while allowing pulmonary translation to account for cell identity, disease phase, and tissue architecture.

## 7. Conclusions

The first generation of HIF-2α therapeutics has demonstrated that oxygen sensing can be manipulated safely and effectively in humans. The challenge now shifts from proving that HIF-2α is druggable to understanding how it should be modulated across the remarkable cellular diversity of the lung. Evidence from developmental biology, human high-altitude genetics, pulmonary vascular disease, fibrosis, emphysema, acute lung injury, epithelial repair, and alveolar immune biology argues that HIF-2α is best understood as a dosage-sensitive regulator of cell state rather than as a uniformly protective or pathogenic factor.

Consequently, the future of pulmonary HIF biology is unlikely to be defined by simply activating or inhibiting the pathway. Instead, progress will depend on matching therapeutic strategy to cell type, disease phase, and biological objective: inhibiting HIF-2α where it sustains pathological remodeling, preserving or restoring it where it supports endothelial or alveolar homeostasis, and targeting downstream circuits where only selected HIF-2α-dependent outputs are pathogenic. Achieving this goal will require close integration between clinicians who can define disease stage and phenotype, and biologists who can resolve the relevant HIF-2α-active cell states using single-cell and spatial technologies, functional genetics, regulatory RNA biology, and emerging delivery platforms.

The complexity of pulmonary HIF-2α biology should therefore guide, rather than deter, the next phase of therapeutic development. If the first generation of HIF-2α drugs established that oxygen sensing is pharmacologically tractable, the next generation will need to determine how to modulate this pathway with the spatial, temporal, and biological precision required for lung disease.

## Figures and Tables

**Table 1:** Human high-altitude genetics as a natural experiment in HIF-pathway tuning.

Population/Locus	Variant or Genetic Feature	Molecular Class	Reported Phenotype or Functional Implication	Relevance to HIF-2α Biology	Key References
Tibetan highlanders/*EPAS1*	Selected *EPAS1* haplotype, including noncoding variants near *EPAS1*	Noncoding regulatory variation; Denisovan-like introgressed haplotype	Associated with lower hemoglobin concentration at altitude and adaptation to chronic hypobaric hypoxia.	Shows that *EPAS1*/HIF-2α pathway output can be tuned by regulatory variation over evolutionary time.	Beall C.M. et al., Proceedings of the National Academy of Sciences of the United States of America, 2010 [26]; Yi X. et al., Science, 2010 [27]; Simonson T.S. et al., Science, 2010 [28]; Huerta-Sánchez E. et al., Nature, 2014 [48]
Tibetan highlanders/*EPAS1* enhancers	Adaptive alleles affecting multiple *EPAS1* enhancers, including hypoxia-sensitive enhancer activity	Enhancer-level regulatory variation	Disruption of *EPAS1* enhancer activity in one or more cell types; allele-specific and oxygen-sensitive effects.	Provides a mechanism by which noncoding variants may alter HIF-2α dosage without changing the HIF-2α protein sequence.	Peng Y. et al., Molecular Biology and Evolution, 2017 [49]; Gray O.A. et al., Science Advances, 2022 [50]
Tibetan highlanders/*EGLN1*	Selected *EGLN1*/PHD2 variants	Upstream oxygen-sensor variation	Associated with high-altitude adaptation; experimental studies suggest altered PHD2 function and ventilatory responses to hypoxia.	Indicates that adaptation can involve tuning of HIF-α degradation and upstream oxygen sensing, not *EPAS1* alone.	Simonson T.S. et al., Science, 2010 [28]; Xiang K. et al., Molecular Biology and Evolution, 2013 [52]; Lorenzo F.R. et al., Nature Genetics, 2014 [53]; Song D. et al., Proceedings of the National Academy of Sciences of the United States of America, 2020 [54]
Andean highlanders/*EPAS1*	rs570553380, encoding HIF-2α H194R	Coding missense variant	Associated with relatively lower hematocrit and protection from excessive erythrocytosis; attenuates canonical HIF-2α target-gene expression in experimental systems.	Demonstrates a distinct, protein-coding route to altered HIF-2α output in a separate high-altitude population.	Eichstaedt C.A. et al., Scientific Reports, 2017 [55]; Jorgensen K. et al., Molecular Biology and Evolution, 2023 [56]; Lawrence E.S. et al., Science Advances, 2024 [57]
Wider human populations/HIF-pathway loci	Variation in *EPAS1*, *EGLN1*, *VHL*, *ARNT*, enhancers, and noncoding RNA loci	Common and rare coding/noncoding variation	Potentially relevant to interindividual differences in hypoxia tolerance, erythropoietic response, pulmonary vascular remodeling, inflammatory repair, or critical illness recovery; incompletely defined.	Provides a rationale for future genotype-to-function studies of HIF-pathway regulation in lung disease and acute hypoxemic illness.	Conceptual extension from high-altitude adaptation studies; requires direct testing in lung and critical-care cohorts

## Data Availability

No new data were created or analyzed in this study. Data sharing is not applicable to this article.

## Supplementary Materials

The following supporting information can be downloaded at https://www.mdpi.com/article/10.3390/biology15211907/s1, Table S1: Completed or reported clinical trials with belzutifan; Table S2: Completed or reported clinical trials with HIF2α inhibitors other than belzutifan; Table S3: Representative SNVs at the *EPAS1* gene that have been characterized in studies for being associated with adaptive phenotypes in high altitudes; Table S4: Representative SNVs at the *EGLN1* gene that have been characterized in studies for being associated with adaptive phenotypes in high altitudes. Table S5: Summary of HIF-2α-relevant cellular compartments, functional effects, evidence levels, and therapeutic implications across non-malignant pulmonary disease states.

## Abbreviations

The following abbreviations are used in this manuscript:

ALI	Acute lung injury
ARDS	Acute respiratory distress syndrome
ARNT	Aryl hydrocarbon receptor nuclear translocator
AT2	Alveolar type II
BMPR2	Bone morphogenetic protein receptor type 2
COPD	Chronic obstructive pulmonary disease
COPD-PH	Chronic obstructive pulmonary disease-associated pulmonary hypertension
CPFE	Combined pulmonary fibrosis and emphysema
CXCL12	C-X-C motif chemokine ligand 12
CXCR4	C-X-C chemokine receptor 4
EGLN/PHD	Egl-9 family hypoxia-inducible factor/prolyl hydroxylase domain enzymes
EGLN1/PHD2	Egl-9 family hypoxia-inducible factor 1/prolyl hydroxylase domain protein 2
ENaC	Epithelial sodium channel
EndMT	Endothelial-to-mesenchymal transition
EPAS1	Endothelial PAS domain protein 1
EPO	Erythropoietin
FIH/HIF1AN	Factor inhibiting HIF-1/Hypoxia-inducible factor 1 subunit alpha inhibitor
FoxM1	Forkhead box M1
H3K4me3	H3 lysine 4 trimethylation
HGF	Hepatocyte growth factor
HIF	Hypoxia-inducible factor
HIF-1α	Hypoxia-inducible factor 1 α
HIF-1β	Hypoxia-inducible factor 1 β
HIF-2α	Hypoxia-inducible factor 2 α
HREs	Hypoxia-response elements
IPF	Idiopathic pulmonary fibrosis
IPF-PH	Pulmonary hypertension associated with idiopathic pulmonary fibrosis
lncRNAs	Long noncoding RNAs
miR-210	microRNA-210
MNT	MAX network transcriptional repressor
mRNA	Messenger ribonucleic acid
MUC5B	Mucin 5B
NFATc2	Nuclear factor of activated T cells c2
p300/CBP	p300/CREB-binding protein
PAH	Pulmonary arterial hypertension
PAS-B	Per-ARNT-Sim B
RNA	Ribonucleic acid
siRNA	Small interfering RNA
VE-cadherin	Vascular endothelial cadherin
VE-PTP	Vascular endothelial protein tyrosine phosphatase
VEGF	Vascular endothelial growth factor
VHL	von Hippel–Lindau
