# Supplementary material for: HIF-2α in Lung Biology and Disease: From Adaptation and Repair to Therapeutic Modulation
Source: Biology (Basel). 2026 Oct 22;15(21):1907. doi: 10.3390/biology15211907 (PMC13761892; doi:10.3390/biology15211907)
Supplement: Supplementary file 1 [file biology-15-01907-s001.zip › biology-4567618-supplementary.pdf]

Table S1. Completed or reported clinical trials with belzutifan.
[up to date as of May 10th, 2026]

**Trial ID**	**Identity of HIF-2α inhibitor**	**Description**	**Type / condition**	**Phase**	**Year of start for trial**	**Current status**
NCT04924075	Belzutifan (PT2977/ MK-6482)	Belzutifan/MK-6482 for the Treatment of Advanced Pheochromocytoma/Paraganglioma (PPGL), Pancreatic Neuroendocrine Tumor (pNET), Von Hippel-Lindau (VHL) Disease-Associated Tumors, Advanced Gastrointestinal Stromal Tumor (wt GIST), or Solid Tumors With HIF-2α Related Genetic Alterations (MK-6482-015)	Multiple	Phase 2	2021/08	Recruiting
NCT04994522	Belzutifan (PT2977/ MK-6482)	A Study of Belzutifan (MK-6482) in Participants With Renal Impairment (MK-6482-021)	End Stage Renal Disease / Renal Impairment	Phase 1	2022/07	Completed
NCT04846920	Belzutifan (PT2977/ MK-6482)	A Study of Belzutifan (MK-6482) in Participants With Advanced Clear Cell Renal Cell Carcinoma (MK-6482-018)	Clear Cell Renal Cell Carcinoma	Phase 1	2021/06	Active, not recruiting
NCT04995484	Belzutifan (PT2977/ MK-6482)	A Study of Belzutifan (MK-6482) in Participants With Hepatic Impairment (MK-6482-020)	Moderate Hepatic Impairment	Phase 1	2022/06	Completed
EUPAS108114	Belzutifan (PT2977/ MK-6482)	Non-interventional post-authorization study of belzutifan in adult patients with von Hippel-Lindau disease-associated renal cell carcinoma, pancreatic neuroendocrine tumor and/or central	Multiple	N/A (PAS study)	2024/07	Ongoing

		nervous system hemangioblastoma (MK-6482-026)				
NCT07167329	Belzutifan (PT2977/ MK-6482)	Real-World Effectiveness and Pharmacogenetics of Belzutifan in VHL Syndrome: The BELIEVE-VHL Trial (BELIEVE-VHL)	VHL Syndrome	Phase 2	2024/01	Recruiting
NCT03445169	Belzutifan (PT2977/ MK-6482)	A Food Effect Study in Healthy Volunteers With Belzutifan (PT2977, MK-6482) Tablets	Feasibility (Healthy)	Phase 1	2018/02	Completed
NCT06677190	Belzutifan (PT2977/ MK-6482)	Belzutifan in Recurrent Clear Cell Carcinoma of Gynecologic Origin	Clear Cell Carcinoma of Gynecologic Origin	Phase 2	2024/12	Recruiting
NCT04489771	Belzutifan (PT2977/ MK-6482)	A Study of Belzutifan (MK-6482) in Participants With Advanced Renal Cell Carcinoma (MK-6482-013)	Advanced Renal Cell Carcinoma	Phase 2	2020/09	Active, not recruiting
NCT06903715	Belzutifan (PT2977/ MK-6482)	A Clinical Study of Belzutifan (MK-6482) Alone or With Phenytoin in Healthy Participants (CA46602/ MK-6482-035)	Feasibility (Healthy)	Phase 1	2025/03	Completed
NCT04627064	Belzutifan (PT2977/ MK-6482)	ABEMA Alone or in COMBO With MK-6482	Clear Cell Renal Cell Carcinoma	Phase 1	2020/12	Completed
NCT05700461	Belzutifan (PT2977/ MK-6482)	Drug Screening Using Novel IMD in Renal Cell Carcinoma	Renal Cell Carcinoma	Phase 1	2024/12	Enrolling by invitation

NCT03401788	Belzutifan (PT2977/ MK-6482)	A Phase 2 Study of Belzutifan (PT2977, MK-6482) for the Treatment of Von Hippel Lindau (VHL) Disease-Associated Renal Cell Carcinoma (RCC) (MK-6482-004)	Multiple	Phase 2	2018/05	Active, not recruiting
NCT05030506	Belzutifan (PT2977/ MK-6482)	A Study of Belzutifan (MK-6482) as Monotherapy and in Combination With Lenvatinib (E7080/MK-7902) With or Without Pembrolizumab (MK-3475) in China Participants With Advanced Renal Cell Carcinoma (MK-6482-010)	Renal Cell Carcinoma	Phase 1	2021/10	Active, not recruiting
NCT02974738	Belzutifan (PT2977/ MK-6482)	A Trial of Belzutifan (PT2977, MK-6482) Tablets In Patients With Advanced Solid Tumors (MK-6482-001)	Multiple	Phase 1	2016/12	Active, not recruiting
NCT06234605	Belzutifan (PT2977/ MK-6482)	A Study of HC-7366 in Combination With Belzutifan (WELIREG™) in Patients With Renal Cell Carcinoma	Renal Cell Carcinoma	Phase 1	2024/04	Recruiting
NCT05468697	Belzutifan (PT2977/ MK-6482)	A Study of Belzutifan (MK-6482) in Combination With Palbociclib Versus Belzutifan Monotherapy in Participants With Advanced Renal Cell Carcinoma (MK-6482-024/LITESPARK-024)	Renal Cell Carcinoma	Phase 1/2	2022/08	Active, not recruiting
NCT07121959	Belzutifan (PT2977/ MK-6482)	A Study of Belzutifan (MK-6482) and Metformin in Healthy Adult Participants (MK-6482-039)	Healthy	Phase 1	2025/08	Completed
NCT07554508	Belzutifan (PT2977/ MK-6482)	Belzutifan VHL RCC HK Prospective Registry	VHL - Von Hippel-Lindau Syndrome	N/A (Observational study)	2026/05	Not yet recruiting

NCT07187778	Belzutifan (PT2977/ MK-6482)	Phase II Trial of Single Agent Belzutifan or Pembrolizumab Versus Combination as Neoadjuvant Therapy in Clear Cell Renal Cell Carcinoma (BLAZE)	Clear Cell Renal Cell Carcinoma	Phase 2	2025/12	Recruiting
NCT03634540	Belzutifan (PT2977/ MK-6482)	A Trial of Belzutifan (PT2977, MK-6482) in Combination With Cabozantinib in Patients With Clear Cell Renal Cell Carcinoma (ccRCC) (MK-6482-003)	Clear Cell Renal Cell Carcinoma	Phase 2	2018/09	Active, not recruiting
NCT07489495	Belzutifan (PT2977/ MK-6482)	A Clinical Study of Belzutifan (MK-6482) and Zanzalintinib in People With Renal Cell Carcinoma (RCC) (LITESPARK-034/LS-034/MK-6482-034)	Renal Cell Carcinoma	Phase 3	2026/04	Recruiting
NCT04195750	Belzutifan (PT2977/ MK-6482)	A Study of Belzutifan (MK-6482) Versus Everolimus in Participants With Advanced Renal Cell Carcinoma (MK-6482-005)	Renal Cell Carcinoma	Phase 3	2020/02	Active, not recruiting
NCT07227402	Belzutifan (PT2977/ MK-6482)	A Clinical Study of Belzutifan and Zanzalintinib in People With Recurrent Kidney Cancer Following Adjuvant Therapy (MK-6482-033) (LITESPARK-033)	Renal Cell Carcinoma	Phase 3	2025/11	Recruiting
NCT05574712	Belzutifan (PT2977/ MK-6482)	A Neoadjuvant Study of Abiraterone Acetate, Leuprolide Acetate, and Belzutifan in Men With Regional Prostate Cancer	Prostate Cancer	Phase 2	2024/01	Withdrawn (Principal Investigator has decided not to activate this trial)
NCT07049926	Belzutifan (PT2977/ MK-6482)	Substudy 03C: A Study of Combination Therapies in Participants With Renal Cell Carcinoma With Recurrent Disease During or After Anti-PD-(L)1 Therapy (MK-3475-03C/KEYMAKER-U03)	Renal Cell Carcinoma	Phase 1b/2	2025/07	Recruiting

NCT07179770	Belzutifan (PT2977/ MK-6482)	Effects of Belzutifan on 89Zr-DFO-girentuximab PET Uptake in Patients With Renal Cell Carcinoma (RCC)	Clear Cell Renal Cell Carcinoma	Phase 1	2025/09	Recruiting
NCT04976634	Belzutifan (PT2977/ MK-6482)	Pembrolizumab Plus Lenvatinib in Combination With Belzutifan in Solid Tumors (MK-6482-016)	Multiple	Phase 2	2021/08	Active, not recruiting
NCT07405164	Belzutifan (PT2977/ MK-6482)	Extension Study for Participants in Studies That Include Belzutifan (MK-6482-043/LITESPARK-043)	Renal Cell Carcinoma, Von Hippel-Lindau Disease	Phase 3	2026/03	Recruiting
NCT05239728	Belzutifan (PT2977/ MK-6482)	A Study of Belzutifan (MK-6482) Plus Pembrolizumab (MK-3475) Versus Placebo Plus Pembrolizumab in Participants With Clear Cell Renal Cell Carcinoma Post Nephrectomy (MK-6482-022)	Clear Cell Renal Cell Carcinoma	Phase 3	2022/03	Active, not recruiting
NCT04586231	Belzutifan (PT2977/ MK-6482)	A Study of Belzutifan (MK-6482) in Combination With Lenvatinib Versus Cabozantinib for Treatment of Renal Cell Carcinoma (MK-6482-011)	Renal Cell Carcinoma	Phase 3	2021/02	Active, not recruiting
NCT07023432	Belzutifan (PT2977/ MK-6482)	Belzutifan's Role in Active Surveillance Versus Treatment for Indolent metastatic Clear Cell Renal Cell Carcinoma (BRAVE-RCC)	Clear Cell Renal Cell Carcinoma	Phase 2	2025/12	Not yet recruiting
NCT06428396	Belzutifan (PT2977/ MK-6482)	Study of Belzutifan (MK-6482) Plus Fulvestrant for ER+/HER2- Metastatic Breast Cancer (MK-6482-029/LITESPARK-029)	Metastatic Breast Cancer	Phase 2	2024/11	Recruiting

NCT04736706	Belzutifan (PT2977/ MK-6482)	A Study of Pembrolizumab (MK-3475) in Combination With Belzutifan (MK-6482) and Lenvatinib (MK-7902), or Pembrolizumab/ Quavonlimab (MK-1308A) in Combination With Lenvatinib, Versus Pembrolizumab and Lenvatinib, for Treatment of Advanced Clear Cell Renal Cell Carcinoma (MK-6482-012)	Clear Cell Renal Cell Carcinoma	Phase 3	2021/04	Active, not recruiting
NCT05899049	Belzutifan (PT2977/ MK-6482)	A Study of Pembrolizumab (MK-3475) in Combination With Belzutifan (MK-6482) and Lenvatinib (MK-7902), or Pembrolizumab/ Quavonlimab (MK-1308A) in Combination With Lenvatinib, vs Pembrolizumab and Lenvatinib, for Treatment of Advanced Clear Cell Renal Cell Carcinoma (MK-6482-012)-China Extension Study	Clear Cell Renal Cell Carcinoma	Phase 3	2022/07	Active, not recruiting
NCT07377526	Belzutifan (PT2977/ MK-6482)	Phase 1/2 Study TROP2 CAREngineered Cord Blood-Derived NK Cells + Belzutifan In Pancreatic Cancer	Pancreatic Cancer	Phase 1/2	2026/07	Not yet recruiting
NCT02861573	Belzutifan (PT2977/ MK-6482)	Study of Pembrolizumab (MK-3475) Combination Therapies in Metastatic Castration-Resistant Prostate Cancer (MK-3475-365/KEYNOTE-365)	Prostate Cancer	Phase 1/2	2016/11	Recruiting
NCT04626518	Belzutifan (PT2977/ MK-6482)	Substudy 03B: A Study of Immune and Targeted Combination Therapies in Participants With Second Line Plus (2L+) Renal Cell Carcinoma (MK-3475-03B/KEYMAKER-U03)	Renal Cell Carcinoma	Phase 1b/2	2020/12	Active, not recruiting
NCT04626479	Belzutifan (PT2977/ MK-6482)	Substudy 03A: A Study of Immune and Targeted Combination Therapies in Participants With First Line (1L) Renal Cell Carcinoma (MK-3475-03A)	Renal Cell Carcinoma	Phase 1b/2	2020/12	Active, not recruiting

NCT0733292 3	Belzutifan (PT2977/ MK-6482)	Predicting HIF-2α Levels in Clear Cell Kidney Cancer Using Machine Learning [Technically, belzutifan is not administered in the study, but rather different parameters and prognostic factors are measured to predict responsiveness to belzutifan]	Clear Cell Renal Cell Carcinoma	N/A (Observati onal)	2024/08	Active, not recruiting

Table S2. Completed or reported clinical trials with HIF2α inhibitors other than belzutifan.
[up to date as of May 10th, 2026]

**Trial ID**	**Identity of HIF-2α inhibitor**	**Description**	**Type / condition**	**Phase**	**Year of start for trial**	**Current status**
NCT07011719	Casdatifan (AB521)	Study of Casdatifan and Cabozantinib Versus Placebo and Cabozantinib in Patients With Advanced Clear Cell Renal Cell Carcinoma	Clear Cell Renal Cell Carcinoma	Phase 3	2025/09	Recruiting
NCT06919991	Casdatifan (AB521)	Drug-Drug Interaction Study of Casdatifan in Healthy Adult Participants (ARC-29)	Feasibility (Healthy)	Phase 1	2025/05	Completed
NCT05117554	Casdatifan (AB521)	Study to Investigate the Safety, Tolerability, and Pharmacokinetic Profile With Oral AB521 in Healthy Volunteers (ARC-14)	Feasibility (Healthy)	Phase 1	2021/11	Completed

NCT05999513	Casdatifan (AB521)	A Relative Bioavailability Study and Food Effect Study of AB521 in Healthy Adult Volunteers (ARC-28)	Feasibility (Healthy)	Phase 1	2023/08	Completed
NCT05536141	Casdatifan (AB521)	Recruiting A Phase 1 Study of AB521 Monotherapy and Combination Therapies in Renal Cell Carcinoma and Other Solid Tumors (ARC-20)	Clear Cell Renal Cell Carcinoma	Phase 1	2022/10	Recruiting
NCT07397611	Casdatifan (AB521)	Pre-NEOSHIFT-RCC: Neoadjuvant HIF-Inhibitor Immunotherapy in RCC	Clear Cell Renal Cell Carcinoma	Phase 2	2026/03	Recruiting
NCT07000149	Casdatifan (AB521)	A Study to Investigate the Efficacy and Safety of Volrustomig ± Casdatifan vs Nivolumab + Ipilimumab as 1L Treatment for Advanced ccRCC (eVOLVE-RCC02)	Clear Cell Renal Cell Carcinoma	Phase 1b/3	2025/07	Active, not recruiting
NCT06191796	Casdatifan (AB521)	Study of Zanzalintinib (XL092) + AB521 and Zanzalintinib + AB521 + Nivolumab in Participants With Advanced Clear Cell Renal Cell Carcinoma (ccRCC) or Other Advanced Solid Tumors (STELLAR-009)	Clear Cell Renal Cell Carcinoma	Phase 1	2024/01	Terminated prior to Phase 2 due to business decision
NCT02553356	PT2385	PT2385-102 Food Effect Study of PT2385 in Healthy Volunteers	Feasibility (Healthy)	Phase 1	2015/09	Completed
NCT04989959	PT2385	[18F]PT2385 PET/CT in Patients With Renal Cell Carcinoma	Renal Cell Carcinoma	Phase 1	2021/08	Recruiting
NCT03216499	PT2385	HIF-2 Alpha Inhibitor PT2385 in Treating Patients With Recurrent Glioblastoma (PT2385)	Glioblastoma	Phase 2	2017/09	Completed

NCT02293980	PT2385	A Phase 1, Dose-Escalation Trial of PT2385 Tablets In Patients With Advanced Clear Cell Renal Cell Carcinoma (MK-3795-001)	Clear Cell Renal Cell Carcinoma	Phase 1	2014/11	Active, not recruiting
NCT03108066	PT2385	MK-3795 (PT2385) for the Treatment of Von Hippel-Lindau Disease-Associated Clear Cell Renal Cell Carcinoma (MK-3795-003)	Clear Cell Renal Cell Carcinoma	Phase 2	2017/04	Completed
NCT04169711	ARO-HIF2 (siRNA)	Study of ARO-HIF2 in Patients With Advanced Clear Cell Renal Cell Carcinoma	Clear Cell Renal Cell Carcinoma	Phase 1b	2020/08	Completed
NCT05119335	NKT2152	A Study of NKT2152, a HIF2α Inhibitor, in Patients With Advanced Clear Cell Renal Cell Carcinoma	Clear Cell Renal Cell Carcinoma	Phase 1/2	2021/10	Terminated as a result of sponsor portfolio reprioritization
NCT05935748	NKT2152	Ph2 Study NKT2152 With Palbociclib & Sasanlimab in Subjects With Advanced Clear Cell Renal Cell Carcinoma (ccRcc)	Clear Cell Renal Cell Carcinoma	Phase 2	2023/07	Terminated as a result of sponsor portfolio reprioritization
NCT04524871	NKT2152	A Study Evaluating the Efficacy and Safety of Multiple Immunotherapy-Based Treatment Combinations in Patients With Advanced Liver Cancers (MORPHEUS-LIVER)	Liver Cancer	Phase 1b/2	2020/11	Recruiting
NCT04895748	DFF332	DFF332 as a Single Agent and in Combination With Everolimus & Immuno-Oncology Agents in Advanced/Relapsed Renal Cancer & Other Malignancies	Advanced/ Relapsed Renal Cancer & Other Malignancies	Phase 1/1b	2021/11	Terminated due to business decision and not related to safety concerns

Table S3. Representative SNVs at the *EPAS1* gene that have been characterized in studies for being associated with adaptive phenotypes in high altitudes (data obtained from 1000 Genomes Project). [up to date as of July 16th, 2026]

**SNV identity**	**Location at/near *EPAS1* (coding region, intron, noncoding, etc.)**	**Major allele**	**Minor Allele**	**Minor Allele Frequency (global)**	**Publication where first described or characterized**
rs4953354	Intron 2, noncoding	A	G	0.22	Beall C.M. et al., Proceedings of the National Academy of Sciences of the United States of America, 2010 [26]
rs13419896	Intron 1, noncoding	G	A	0.18	Beall C.M. et al., Proceedings of the National Academy of Sciences of the United States of America, 2010 [26]
rs149594770	Intron 1, noncoding	T	A	0.01	Peng Y. et al., Molecular Biology and Evolution, 2017 [49]
rs570553380	Exon 6, coding (H194R)	A	G	< 0.01	Eichstaedt C.A. et al., Scientific Reports, 2017 [55]

Table S4. Representative SNVs at the *EGLN1* gene that have been characterized in studies for being associated with adaptive phenotypes in high altitudes (data obtained from 1000 Genomes Project). [up to date as of July 16th, 2026]

**SNV identity**	**Location at/near *EGLN1* (coding region, intron, noncoding, etc.)**	**Major allele**	**Minor Allele**	**Minor Allele Frequency (global)**	**Publication where first described or characterized**

rs186996510	Exon 1, coding (D4E)	G	C	< 0.01	Xiang K. et al., Molecular Biology and Evolution, 2013 [52];<br>Lorenzo et al., Nature Genetics 2014 [53]
rs12097901	Exon 1, coding (C127S)	C	G	0.26	Xiang K. et al., Molecular Biology and Evolution, 2013 [52];<br>Lorenzo F.R. et al., Nature Genetics, 2014 [53]

Table S5. Summary of HIF-2α-relevant cellular compartments, functional effects, evidence levels, and therapeutic implications across non-malignant pulmonary disease states.

Disease / phenotype	Principal HIF-2α-relevant compartment(s)	Functional role	HIF-2α specificity	Evidence level	Potential therapeutic implication	Representative references
**Pulmonary arterial hypertension (PAH)**	Pulmonary endothelium, pericytes; less clearly smooth muscle and immune compartments		Strong HIF-2α-specific evidence in endothelial and pericyte compartments; broader HIF signaling contribution in other vascular compartments	Human tissue/cells; animal genetic and pharmacologic models	HIF-2α inhibition may be beneficial where pathogenic HIF-2α-active vascular states predominate	Cowburn, A.S. et al., Proceedings of the National Academy of Sciences, 2016 [19]; Kim, H. et al., EMBO Reports, 2024 [20]; Tang, H. et al., American Journal of Physiology-Lung Cellular and Molecular Physiology, 2018 [68]
**Pulmonary fibrosis / IPF**	Fibroblasts and aberrant epithelial repair states	Supports fibroblast proliferation and may promote persistent maladaptive epithelial repair	Isoform-specific evidence exists in human fibroblasts and epithelial repair models; broader fibrosis literature also includes HIF-	Human tissue association; human in-vitro/organoid studies; animal genetic/pharm	Selected HIF-2α-active fibrotic states may be candidates for inhibition; therapeutic efficacy in	McCall, A.S. et al., Science Translational Medicine, 2025 [23]; Bodempudi, V. et al., American Journal of Physiology-Lung Cellular and Molecular

			1α/general HIF effects	acologic models	human IPF remains unproven	Physiology, 2014 [81]; Senavirathna, L.K. et al., Scientific Reports, 2018 [82]
**COPD / emphysema**	Pulmonary endothelium	Supports pulmonary microvascular maintenance and epithelial–endothelial trophic signaling	Direct endothelial HIF-2α loss-of-function evidence	Human lung tissue; endothelial genetic mouse models	Preservation or restoration of endothelial HIF-2α may be preferable to inhibition in emphysema-predominant disease	Pasupneti, S. et al., American Journal of Respiratory and Critical Care Medicine, 2020 [22]; Jiang, X. et al., Circulation, 2019 [83]; Myronenko, O. et al., European Respiratory Society, 2023 [84]
**IPF-PH / CPFE / COPD-PH**	Mixed vascular, fibrotic, and emphysematous compartments	Protective and pathogenic HIF-2α programs may coexist within different regions or disease phases	Compartment-dependent; extrapolated in part from component disease mechanisms	Human phenotypic data plus mechanistic evidence from PAH, fibrosis, and emphysema studies	Requires phenotype-, stage-, and biomarker-guided modulation rather than uniform inhibition	Pasupneti, S. et al., American Journal of Respiratory and Critical Care Medicine, 2020 [22]; McCall, A.S. et al., Science Translational Medicine, 2025 [23]; Cottin, V. et al., European Respiratory Society, 2009 [87]
**ALI / ARDS**	Pulmonary endothelium	HIF-2α supports endothelial barrier integrity during acute injury; HIF-1α contributes more prominently to proliferative endothelial repair	Experimentally differentiated HIF-1α and HIF-2α functions	Animal genetic/injury models	Timing and disease phase are critical; HIF-2α inhibition during barrier-protective phases could be harmful	Gong, H. et al., Journal of Clinical Investigation, 2015 [24]; Huang, X. et al., The American Journal of Pathology, 2019 [90]
**Airway / muco-**	Airway and alveolar epithelial progenitors	Hypoxia/HIF signaling promotes airway-like	Disease-specific HIF-2α evidence remains limited;	Human organoids;	Insufficient evidence for HIF-2α-specific	Dong, Z. et al., Cell Stem Cell, 2025 [64]; Polosukhin, V.V. et al.,

**obstructive disease**		differentiation; HIF-2α contributes to basal-lineage bias	several mucus-related phenotypes involve HIF-1α or combined HIF activity	experimental airway models	therapeutic targeting at present	The Journal of Pathology, 2011 [85]; Mikami, Y. et al., Science Translational Medicine, 2023 [86]
**Alveolar macrophage dysfunction**	Alveolar macrophages	Persistent HIF-2α activity disrupts fatty-acid oxidation and surfactant clearance	HIF-1α and HIF-2α regulate overlapping but experimentally distinguishable programs	Mouse genetic models	Potential future target, but disease-specific translational evidence remains limited	Priego, E. et al., Cell Reports 2026 [25]
